# Clinical and genetic Rett syndrome variants are defined by stable electrophysiological profiles

**DOI:** 10.1186/s12887-018-1304-7

**Published:** 2018-10-19

**Authors:** Conor Keogh, Giorgio Pini, Adam H. Dyer, Stefania Bigoni, Pietro DiMarco, Ilaria Gemo, Richard Reilly, Daniela Tropea

**Affiliations:** 1 School of Medicine, Trinity College Dublin, 152-160 Pearse Street, Dublin 2, Ireland; 2 Tuscany Rett Center, Ospedale Versilia, 55043 Lido di Camaiore, Italy; 3 Medical Genetic Unit, Ferrara University Hospital, Ferrara, Italy; 4 Trinity Centre for Bioengineering, Trinity College Dublin, Dublin 2, Ireland; 5 Neuropsychiatric Genetics, Trinity Centre for Health Sciences, St. James’s Hospital, D8 Dublin, Ireland; 6 Trinity College Institute of Neuroscience (TCIN), Lloyd Building, Trinity College Dublin, Dublin 2, Ireland

**Keywords:** Rett syndrome, MeCP2, CDKL5, EEG, Network

## Abstract

**Background:**

Rett Syndrome (RTT) is a complex neurodevelopmental disorder, frequently associated with epilepsy. Despite increasing recognition of the clinical heterogeneity of RTT and its variants (e.g Classical, Hanefeld and PSV(Preserved Speech Variant)), the link between causative mutations and observed clinical phenotypes remains unclear. Quantitative analysis of electroencephalogram (EEG) recordings may further elucidate important differences between the different clinical and genetic forms of RTT.

**Methods:**

Using a large cohort (*n* = 42) of RTT patients, we analysed the electrophysiological profiles of RTT variants (genetic and clinical) in addition to epilepsy status (no epilepsy/treatment-responsive epilepsy/treatment-resistant epilepsy). The distribution of spectral power and inter-electrode coherence measures were derived from continuous resting-state EEG recordings.

**Results:**

RTT genetic variants (*MeCP2/CDLK5*) were characterised by significant differences in network architecture on comparing first principal components of inter-electrode coherence across all frequency bands (*p* < 0.0001). Greater coherence in occipital and temporal pairs were seen in *MeCP2* vs *CDLK5* variants, the main drivers in between group differences. Similarly, clinical phenotypes (Classical RTT/Hanefeld/PSV) demonstrated significant differences in network architecture (*p* < 0.0001). Right tempero-parietal connectivity was found to differ between groups (*p* = 0.04), with greatest coherence in the Classical RTT phenotype. PSV demonstrated a significant difference in left-sided parieto-occipital coherence (*p* = 0.026). Whilst overall power decreased over time, there were no difference in asymmetry and inter-electrode coherence profiles over time. There was a significant difference in asymmetry in the overall power spectra between epilepsy groups (*p* = 0.04) in addition to occipital asymmetry across all frequency bands. Significant differences in network architecture were also seen across epilepsy groups (*p* = 0.044).

**Conclusions:**

Genetic and clinical variants of RTT are characterised by discrete patterns of inter-electrode coherence and network architecture which remain stable over time. Further, hemispheric distribution of spectral power and measures of network dysfunction are associated with epilepsy status and treatment responsiveness. These findings support the role of discrete EEG profiles as non-invasive biomarkers in RTT and its genetic/clinical variants.

**Electronic supplementary material:**

The online version of this article (10.1186/s12887-018-1304-7) contains supplementary material, which is available to authorized users.

## Background

Rett Syndrome (RTT) is a rare neurodevelopmental disorder affecting 1 in 10,000–20,000 live female births [1–3]. Patients with RTT typically undergo normal development for the first 18 months of life, followed by a period of stagnation and subsequent regression in cognitive and psychomotor abilities. The disorder is characterised by several well-defined stages consisting of: (I) early onset stagnation, (II) developmental regression, (III) pseudostationary period and (IV) late motor deterioration [4]. Further, the clinical picture in RTT is complicated by associated clinical problems, most notably a high incidence of epilepsy which is frequently resistant to treatment, as well as gastro-intestinal problems, gait disturbance, scoliosis, osteopenia and cardiorespiratory dysfunction [4].

Increasing attention has been drawn to this rare disorder as a consequence of the discovery of causative mutations in *MeCP2* (Methyl CpG Binding Protein 2), a gene involved in brain development, neuronal structure and synaptic function [5, 6], in the majority (80–85%) of cases [7]. Further, a rarer variant of Rett Syndrome characterised by mutations in the *CDKL5* (Cyclin-Dependent Kinase-Like 5) gene, a regulator of *MeCP2* which also has important roles in brain development and neuronal maturation [8], suggests a common underlying mechanism related to abnormalities in synapse formation in Rett Syndrome.

Whilst the molecular underpinnings of RTT suggest a single common pathway of abnormal synaptic regulation during development, RTT is increasingly recognised as a clinically heterogenous disorder with widely varying clinical phenotypes [4]. Among the best characterised are the Hanefeld variant, closely linked to mutations in *CDKL5*; these patients may not show the same development and regression pattern with which is characteristic of the classical RTT phenotype, but have a pathognomonic early onset of seizures [9, 10]. The Preserved Speech Variant (PSV), or “Zappella” variant, which is characterised by relatively preserved speech in addition to a less severe clinical picture [11, 12], associated with mutations in the same gene as the Classic variant (*MeCP2*), further highlights the clinical heterogeneity observed, even in the context of mutations in the same causative gene.

Our increasing knowledge of the genetic underpinnings of RTT variants is therefore paired with a relatively poor understanding of the specific neuropathological changes brought about by these genetic abnormalities, and how these reflect the variability observed at the clinical level. Greater elucidation of the nature of these changes may offer insights into the pathological mechanisms underlying RTT variants, as well as offering potential biomarkers for diagnosis, classification and prognostication. Given the lack of correspondence between the genetics and the clinical presentation, it is predicted that specific patterns of abnormality in central nervous system structure and function may be responsible for the differences observed in the separate phenotypes. A suitable “endophenotype”, which may act as an intermediary between the underlying molecular pathology and clinical presentation, may therefore be a characterisation of nervous system functioning using the analysis of quantitative electrophysiological data.

While initial EEG analysis have supported the presence of electrophysiological abnormalities in RTT, a quantitative analysis of how continuous resting-state electrophysiological features relate to specific genetic and phenotypic variants of RTT has been absent [13–17]. A thorough analysis of the differences between these subtypes may therefore offer novel insight into the mediation of clinical phenotypes and how these relate to the underlying genetics.

Notably, EEG metrics have proven to be a valuable tool to understand the pathophysiology of brain dysfunction in related disorders. This is the case for Autism Spectrum Disorder (ASD), which has been the subject of many electrophysiological studies as reviewed elsewhere [18–20]. In studies examining cortical connectivity in ASD, robust patterns of network-level dysfunction have been demonstrated by several authors [21, 22]. Despite evidence that many genes related to ASD have pervasive roles in neurodevelopment, synaptic formation and maintenance in a similar manner to those underlying RTT and related subtypes [23], whether abnormalities at the network level are seen in RTT and its subtypes has never been explored. Such approaches offer the potential to investigate whether genetic and clinical subtypes of RTT are associated with specific abnormalities in network-level architecture, which may offer greater insight into the nature and classification of these groups.

The importance of electrophysiological abnormalities is further underlined by high rates of co-morbid epilepsy in RTT. The associated epilepsy is frequently resistant to treatment and represents a significant clinical problem in this patient cohort. In a large study of RTT patients, epilepsy was present in two-thirds (64.2%) of patients with all-type RTT, with treatment-resistant epilepsy in under one fifth (17.2%) [4]. Epilepsy is present in all of those with the Hanefeld variant of RTT [4]. It follows from the clinical differences in seizure presence and response to treatment that there may be underlying electrophysiological differences within the different RTT phenotypes. The relationship between epilepsy status and electrophysiological characteristics in RTT has, however, never been investigated.

In the present study we therefore characterised the electrophysiological features of the major genetic and clinical subtypes of RTT. In addition, we examined whether differences in these features were associated with epilepsy status and treatment responsiveness in this patient group. Our analysis demonstrates that RTT variants are characterised by specific abnormalities in EEG parameters which are stable across time, further parsing the neurobiological and clinical heterogeneity in these increasingly characterised subgroups, and that EEG measures have the potential to act as endophenotypes in these disorders, with a potential role in diagnosis, classification and prognostication.

## Methods

### Subject recruitment

Patients were recruited from the Tuscany Rett Centre, Italy. All experiments were undertaken in accordance with the Declaration of Helsinki and approved by the Ethical Committee: approval ID: 12720. Patients’ families gave consent and for collection and use of the data for scientific purposes. 42 patients were recruited, with a mean age of 7.69 +/− 5.22 years. Further details on patient demographics are available anonymously in Additional file 1: Appendix 1 and Table S1.

### Data collection

Clinical, genetic and electrophysiological data was recorded for each participant. In any case where the relevant clinical or genetic information was not available for a specific patient, that patient was excluded from electrophysiological analysis. Clinical data was available for 35, genetic data for 40 and epilepsy status for 42 patients.

#### Clinical characterisation

Clinical phenotype was recorded for each patient based on their presentation. This was divided into the common Classic phenotype, the rarer, more severe, Hanefeld variant, and the rare but milder Preserved Speech Variant (PSV) [9]. Epilepsy status was also measured for each patient, recorded as No Epilepsy, Epilepsy or Treatment Resistant Epilepsy based on *(1)* whether there was clinical evidence of epilepsy and *(2)* whether epilepsy responded to medical management.

#### Genetic characterisation

Causative mutations were identified for each patient. These were recorded based on the gene affected: *MeCP2*, the gene most commonly implicated in Rett Syndrome [7], and *CDKL5*, a more rarely affected gene associated with more severe clinical presentations [8].

#### Electrophysiological characterisation

Electroencephalographic (EEG) data was recorded using an eight-electrode montage, with electrodes in frontal, temporal, parietal and occipital locations bilaterally (see Additional file 1: Figure S1, for a schematic of electrode montage). Reference electrodes were placed on the mastoid processes. The ground electrode was placed in position FpZ (midline sagittal plane). Recordings were made from awake subjects seated for a minimum of 20 min continuously at rest (range: 20 min to 204 to minutes, mean 59 min). Recordings were sampled at a rate of 128 Hz. All recordings were carried out under the same testing conditions.

### Preprocessing

The first and last five minutes of each recording were discarded to reduce contamination with movement artefacts. Data were then visually inspected to verify recording quality. Data were split into ten-minute epochs and an automated artefact rejection algorithm run. All remaining epochs were manually examined. The first sufficiently artefact-free epoch was extracted from each recording for analysis in order to ensure inclusion of stationary signals without wide epileptic abnormalities. In order to ensure that these results were not impacted by undetected artefacts, all analyses were repeated using a series of combinations of shorted epochs which were then averaged (see Additional file 1: Appendix 2: Epoch Length). Results were consistent across each iteration, and so the results of the ten-minute epochs are presented here.

**Fig. 1:**
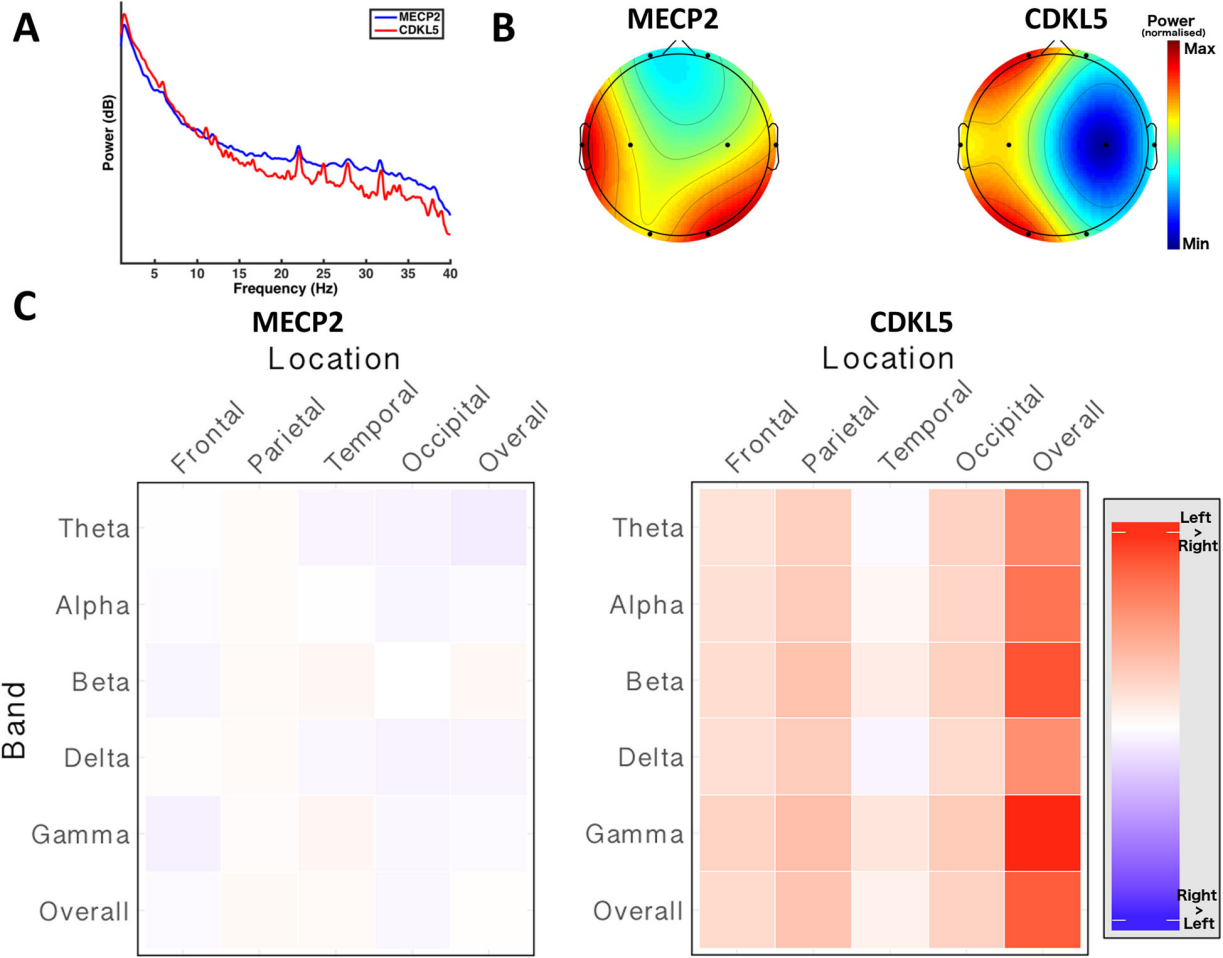
Spectral power profile of MECP2 and CDKL5 gene variants. All subplots depicting a statistical comparison significant at a *p* value of < 0.05 are marked with an asterisk (*). **a** Overall power spectra between 1 Hz and 40 Hz, MECP2 (*n* = 36, blue) and CDKL5 (*n* = 4, red) variants. The between-groups difference for the overall spectra was not statistically significant (Mann-Whitney U test; *p* > 0.05). **b** Head plots demonstrate the spatial distribution of overall spectral power in MECP2 (*left*) and CDKL5 (*right*) genetic variants. Dots represent electrode locations. Colour maps show relative power of the overall spectrum interpolated between electrodes. Colour maps were calculated using the maximum (red) and minimum (blue) across the entire population and applied to both groups, allowing direct comparison of power distribution. The differences in power at each individual electrode were not statistically significant (Mann-Whitney U test; *p* > 0.05). **c** Profile of hemispheric asymmetry at each frequency band by electrode location for MECP2 (*left*) and CDKL5 (*right*). Each column represents a scalp location. Each row represents a frequency band. Cell colour is determined by the asymmetry of the corresponding band at the corresponding location, calculated by subtracting the power in that band at that location on one side from the other. Red indicates greater power in the left hemisphere, blue indicates greater power in the right hemisphere, and intensity indicates the magnitude of the asymmetry. The MECP2 group do not demonstrate an obvious pattern of hemispheric asymmetry, while the CDKL5 group demonstrate a tendency towards asymmetry favouring the left hemisphere, though these differences are not statistically significant (Mann-Whitney U test; *p* > 0.05)

All recordings were transformed to a common eight-electrode montage (see Fig. 1, Additional file 1), with any additional channels present in individual subjects discarded.

Data were baseline corrected by subtraction of the mean of all channels, re-referenced to the average of all scalp channels and digitally filtered offline at 1 Hz - 50 Hz.

### Feature extraction

In order to characterise differences in electrophysiology within Rett Syndrome subtypes, a profile of electrophysiological features was derived using custom MatLab scripts.

#### Spectral power

Overall power spectra were calculated over the full ten minute epochs by Fourier Transform, allowing gross assessment of differences in the 1 - 50 Hz range and evaluation of the spatial distribution of the overall power.

Individual frequency bands were assessed by isolating theta (4 Hz – 8 Hz), alpha (8 Hz – 12 Hz), beta (12 Hz – 30 Hz), delta (0.5 Hz – 4 Hz) and gamma (> 30 Hz) bands [24]. This allowed characterisation of the distribution of activity at specific oscillatory frequencies across the scalp.

Overall power was assessed using the mean of the individual channel spectra. This was measured in absolute power (dB). Power in individual bands was normalised with respect to overall power to give a measure of relative power. Each of these results represents the mean of the power measures within that band.

#### Asymmetry

Relative activity of each hemisphere was assessed in order to evaluate whether there was a marked dominance of one hemisphere, suggestive of abnormalities in distribution of activity. This was calculated by subtracting the overall power in the right hemisphere from power in the left hemisphere.

Profiles of hemispheric symmetry were further characterised by examining the asymmetry in overall power between corresponding electrode pairs, allowing evaluation of the distribution of power in frontal, temporal, parietal and occipital regions specifically. Asymmetry profiles were then derived for each frequency band, allowing investigation of hemispheric dominance within individual bands.

#### Network measures

Interactions between electrodes were evaluated in order to provide an assessment of network function [25]. Measures of inter-electrode coherence were derived for each electrode pair through calculation of the cross-spectrum of two channels normalised by the power spectra of both channels, repeated for each unique pair:

$$ \mathrm{C}\left(\upomega \right)=\frac{{\mathrm{S}}_{\mathrm{xy}}{\left(\upomega \right)}^2}{{\mathrm{S}}_{\mathrm{xx}}\left(\upomega \right)\ {\mathrm{S}}_{\mathrm{yy}}\left(\upomega \right)} $$

This provides a measure of the phase stability between the signals at each electrode, with high degrees of inter-electrode coherence indicating functional connectivity between the two electrodes. Coherence is measured on a scale of 0 (no coherence) to 1 (full coherence).

In order to evaluate overall differences in network architecture between groups while avoiding large numbers of statistical comparisons, dimensionality reduction was carried out using a principal component analysis performed on the inter-electrode coherence measures for each subject. The first principal components, accounting for the highest degree of variance within the coherence measures, were then compared between groups to evaluate differences in overall networks.

Higher-order network architecture was visualised by deriving covariance matrices of inter-electrode coherence measures.

### Longitudinal analysis

We followed up nine subjects (mean age 6.33 +/− 4.33 years) at an interval of 10–14 months and re-assessed their electrophysiological profiles to evaluate whether the observed patterns were stable features of RTT subtypes.

This group consisted of 7 patients with *MeCP2* mutations (all Classic phenotype) and 2 with *CDKL5* mutations; analysis of the *MeCP2* subgroup demonstrated similar results to the overall group, while the small number of *CDKL5* subjects prevented a subgroup analysis of this group. As the results of the *MeCP2* group and the pooled group followed the same pattern, the results for the overall group are presented.

### Statistical comparisons

As a consequence of the relatively small numbers of the rare variants of Rett Syndrome, nonparametric statistical tests were used throughout to avoid assumptions of normality within the small subgroups. Kruskal-Wallis H testing was used for multi-group comparisons, and Mann-Whitney U tests were used for pairwise comparisons. Wilcoxon signed-rank tests were used for comparison of paired data in longitudinal analyses. All tests were two-tailed.

In order to avoid large numbers of statistical comparisons, analysis was initially restricted to overall data, or to measures derived from dimensionality reduction methods (principal component analysis of inter-electrode coherence measures), with further exploration of subgroups based on initial evaluation of overall measures.

All results are reported as mean +/− standard deviation. All data processing was performed blinded to patient characteristics.

### Subsampling

As the patient population is dominated by the more common variants of Rett Syndrome, a subsampling method was employed to re-analyse the features between groups in order to verify that results were not skewed by the uneven distribution of Rett subtypes. Samples of four subjects were drawn from the large *MeCP2* group and compared to the *CDKL5* group across all main features compared. This was repeated with 15 randomly drawn subsamples and the results analysed to ensure consistency.

These results demonstrated consistency across multiple subsamples, and were consistent with the results obtained using the full population, indicating that the results were not biased by the uneven distribution of Rett subtypes. As a result, the results of comparisons using the larger overall population are presented here. The full results of the subsampled analysis can be found in Additional file 1: Appendix 3: Subsampling Results.

### Experimental design

Patient recruitments and clinical analyses were performed by personnel who were not involved in EEG analysis. Processing and feature extraction algorithms were applied to all recordings without consideration of presentation, genetics or other clinical parameters.

## Results

### Electrophysiological profiles of genetic variants

In order to assess whether the effects of different pathogenic mutations were mediated through distinct patterns of electrophysiological dysfunction, we compared the electrophysiological profiles of groups with confirmed mutations in *MeCP2* (*n* = 36) and *CDKL5* (*n* = 4).

#### Spectral power profile

The power spectra of the *MeCP2* and *CDKL5* groups between 1 Hz and 40 Hz were compared (Fig. 1).

We found no significant difference in the overall power spectra of the genetic variants (*MeCP2*: 2.5 +/− 8.57, *CDKL5*: 1.75 +/− 8.63; *p* = 1, Mann-Whitney U test), indicating no gross difference in the overall electrical activity of the cortex between groups (Fig. 1a).

We then analysed the distribution of the overall power across the eight electrodes (Fig. 1b). The *MeCP2* variant demonstrated a pattern of high relative power in temporal and occipital regions bilaterally with relatively low power in frontal regions. The *CDKL5* variant show a pattern of diffuse high power in the left hemisphere, with low power throughout the right hemisphere. Quantitative assessment of these differences in distribution were not statistically significant (*p* > 0.05, Mann-Whitney U test; see Additional file 1: Table S2, for results of comparisons of overall spectrum at each electrode location).

The distribution of power within individual EEG bands was investigated (Additional file 1: Figure S2). We found that the patterns suggested by the overall power spectrum were evident within individual frequency bands (see Additional file 1: Table S3, for results of comparisons of each frequency band).

We then measured hemispheric asymmetry to evaluate differences in the balance of cortical functioning between groups (Fig. 1c). The *MeCP2* group does not exhibit a major pattern of hemispheric dominance, while the *CDKL5* group trends toward left hemispheric dominance in all areas and across all bands. This trend, however, was not statistically significant (*MeCP2*: 0.11 +/− 16.00 vs. 11.92 +/− 26.69; *p* = 0.60, Mann-Whitney U test). (see Additional file 1: Table S4, for comparisons of asymmetry at each electrode location).

#### Network measures

In order to assess whether different pathogenic mutations resulted in distinct patterns of network dysfunction, we analysed patterns of inter-electrode coherence.

We compared the first principal components of inter-electrode coherence measures across all frequency bands, and we found a statistically significant difference in network architecture between genetic variants (*p* < 0.0001, Mann-Whitney U test), suggesting that genetic variants of Rett Syndrome are associated with discrete patterns of network dysfunction. A comparison restricted solely to measures within the overall spectrum was also statistically significant (*p* = 0.026, Mann-Whitney U test). A breakdown of the percentage of the total variance explained by each of the first five principal components for each group can be found in Additional file 1: Table S5.

**Fig. 2:**
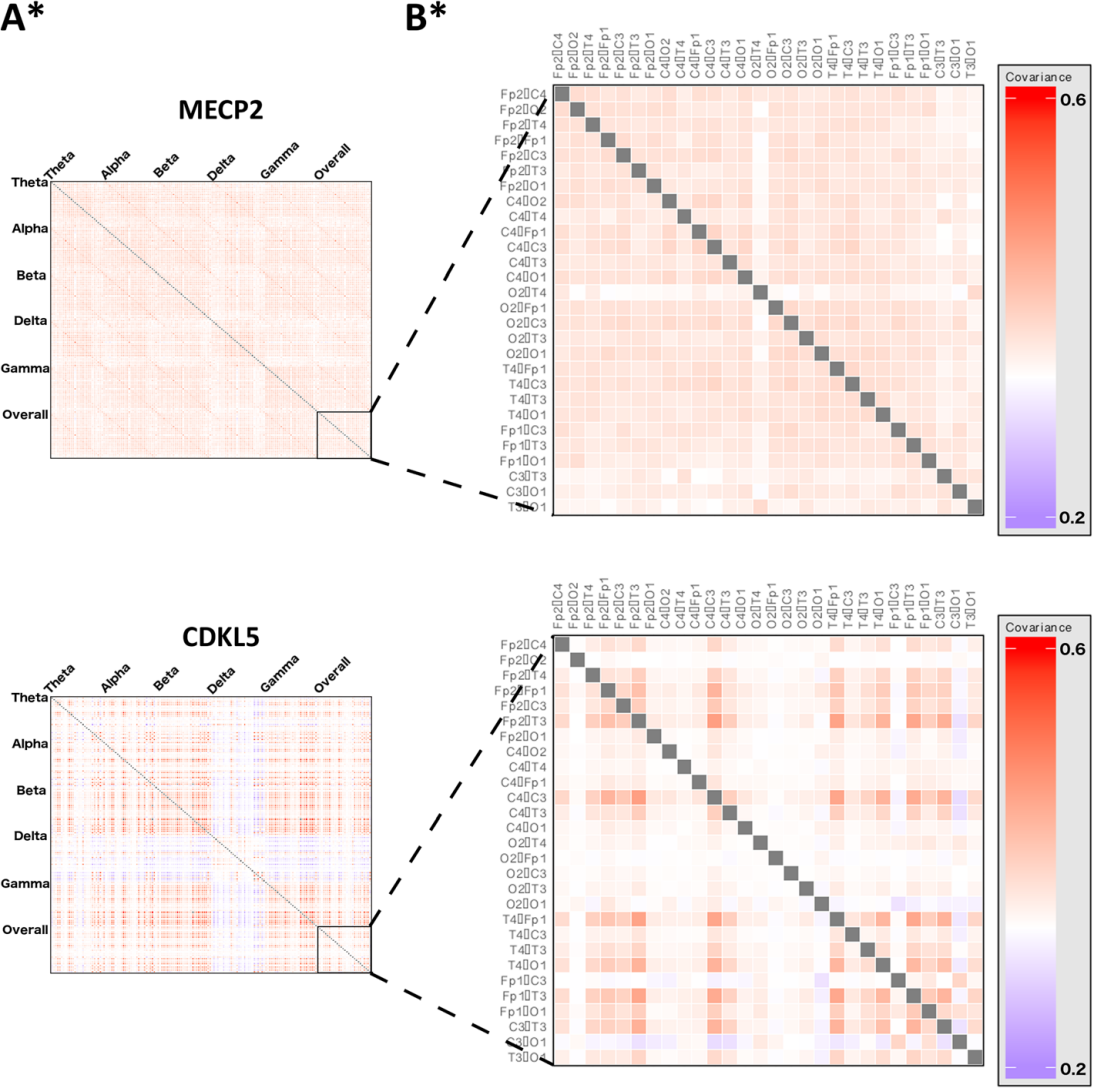
MECP2 and CDKL5 gene variants differ in network architecture. All subplots depicting a statistical comparison significant at a *p* value of < 0.05 are marked with an asterisk (*). **a** Covariance matrices of inter-electrode coherence measurements for MECP2 (n = 36) and CDKL5 (*n* = 4) groups, allowing visualisation of higher-order network function. Each row and each column represent a pair of electrodes. These are arranged into blocks along the axes, with measures for each electrode pair at all frequency bands and across the overall spectrum. The intensity of each cell represents the covariance between activity in the corresponding electrode pairs at the corresponding frequency band. Visualisation suggests differences in network activities; comparison of first principal components indicated a statistically significant difference between groups (*p* < 0.0001, Mann-Whitney U test), indicating that there are differences in network architecture between the genetic subtypes. The MECP2 group primarily shows a pattern of low covariance in networks involving left-sided occipital and temporal electrode pairs, as well as reduced involvement of right-sided occipito-temporal pairs. The CDKL5 network architecture is visually different, primarily distinguished by very low covariance cross all pairs in delta band, including cross-frequency, indicating abnormalities in network function within this frequency range, as well as very low network involvement of right occipital and parietal regions. **b** Subplots of overall covariance matrices in A, showing only covariance between electrode pairs over the whole power spectrum. Each row and each column represents an electrode pair as labelled. Closer examination of covariance within the overall spectrum suggests that differences seen across the whole matrix are still evident within the overall spectrum alone

We then evaluated the overall network architecture for each group by deriving a covariance matrix of inter-electrode coherence measures (Fig. 2a). This representation demonstrates the overall patterns of network activity between and across frequency bands.

We explored the nature of network-level differences between groups. The spatial distribution of electrode pairs found to have a statistically significant difference in coherence in the overall power spectrum between groups (using a threshold of *p* < 0.05, Mann-Whitney U test) and a profile of the direction and magnitude of these differences is illustrated in Fig. 2.

We identified occipital (O1 & O2 pairs) and temporal (T3 & T4 pairs) as the primary drivers of differences between groups (Fig. 2a), suggesting that differences in occipito-temporal network function may result from differences in the underlying causative mutation (see Additional file 1: Table S6, for coherence measures between each electrode pair). The *MeCP2* variant showed greater coherence in each of these connections (Fig. 2b), suggesting that this mutation is associated with greater network function in these areas, while the *CDKL5* variant has less activity in these networks.

### Electrophysiological profiles of phenotypic variants

In order to assess whether differences in network function evident between genetic variants were conserved at the phenotypic level, acting as a bridge between genetic abnormalities and the observed clinical phenotype, we evaluated differences in electrophysiological profile between Classic (*n* = 26), Hanefeld (*n* = 4) and PSV (*n* = 5) phenotypic groups.

#### Spectral power profile

**Fig. 3:**
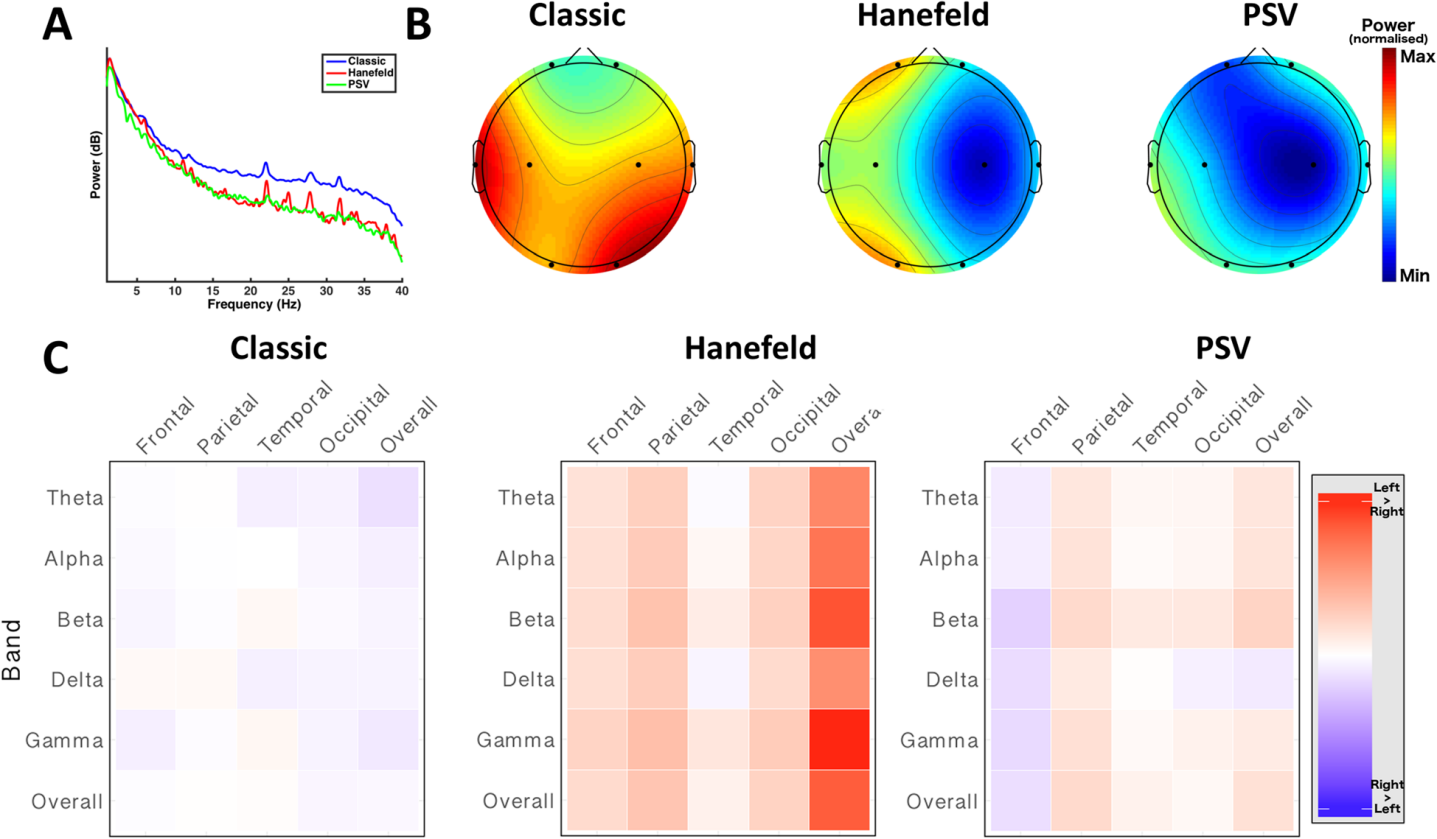
Spectral power profiles of phenotypic variants of Rett Syndrome. All subplots depicting a statistical comparison significant at a p value of < 0.05 are marked with an asterisk (*). **a** Overall power spectra between 1 Hz and 40 Hz for Classic (*n* = 26, blue), Hanefeld (n = 4, red) and PSV (*n* = 5, green) phenotypes. The Classic group shows a tendency towards higher power in the higher range of frequencies, though the differences in overall spectra between phenotypic groups were not statistically significant (Kruskal-Wallis H test; p > 0.05). **b** Head plots demonstrate the spatial distribution of overall spectral power in Classic (*left*), Hanefeld (*middle*) and PSV (*right*) phenotypes. Dots represent electrode locations. Colour maps show relative power of the overall spectrum interpolated between electrodes. Colour maps were calculated using the maximum (red) and minimum (blue) across the entire population and applied to all groups, allowing direct comparison of power distribution between groups. The differences in power at each individual electrode were not statistically significant (Kruskal-Wallis H test; *p* > 0.05). **c** Profile of hemispheric asymmetry at each frequency band by electrode location for Classic (*left*), Hanefeld (*middle*) and PSV (*right*) phenotypes. Each column represents a scalp location. Each row represents a frequency band. Cell colour is determined by the asymmetry of the corresponding band at the corresponding location, calculated by subtracting the power in that band at that location on one side from the other. Red indicates greater power in the left hemisphere, blue indicates greater power in the right hemisphere, and intensity indicates the magnitude of the asymmetry. The Classic group does not demonstrate an obvious pattern of hemispheric asymmetry, while the PSV and, to a greater extent, the Hanefeld group demonstrate a tendency towards asymmetry favouring the left hemisphere, though these differences are not statistically significant when all groups are compared (Kruskal-Wallis H test; *p* > 0.05)

We characterized the power spectra of the Classic, Hanefeld and PSV phenotypic groups between 1 Hz and 50 Hz (Fig. 3).

The overall power spectra of the phenotypic variants (Fig. 3a) demonstrates a trend towards higher power in the Classic group at higher frequencies, though we found that this difference was not statistically significant (Classic: 4.27 +/− 8.80, Hanefeld: 1.75 +/− 8.63, PSV: 0.71 +/− 4.42; *p* = 0.73, Kruskal-Wallis H test).

The distribution of the overall power across the scalp suggested gross differences in the activity of specific brain regions between phenotypes (Fig. 3b). However, quantitative assessment of these difference in distribution by comparisons of overall power between corresponding electrode sites was not statistically significant (*p* > 0.05, Kruskal-Wallis H test; see Additional file 1: Table S7, for results of comparisons of overall spectrum at each electrode location).

A similar analysis within individual bands demonstrated similar patterns to that observed for the overall spectrum (Additional file 1: Figure S5). (see Additional file 1: Table S8, for results of comparisons of each frequency band).

We then measured hemispheric asymmetry between groups (Fig. 3c). The Classic group does not exhibit a major pattern of hemispheric dominance, while the Hanefeld group trends toward left hemispheric dominance. The PSV group also demonstrates a less marked trend towards left hemispheric dominance. Comparison of differences in overall asymmetry between groups was not statistically significant, however (Classic: − 0.48 +/− 17.38, Hanefeld: 11.92 +/− 26.69, PSV: 2.38 +/− 17.39; *p* = 0.87, Kruskal-Wallis H test). (see Additional file 1: Table S9, for comparisons of asymmetry at at each electrode location).

#### Network measures

We compared profiles of network-level activity between phenotypes. Comparison of first principal components of inter-electrode coherence measures across all frequency bands demonstrated a statistically significant difference in network architecture between phenotypes (*p* < 0.0001, Kruskal-Wallis H test), suggesting a role for network dysfunction in the mediation of observed clinical subtypes. A breakdown of the percentage of the total variance explained by each of the first five principal components for each group can be found in Additional file 1: Table S10.

**Fig. 4:**
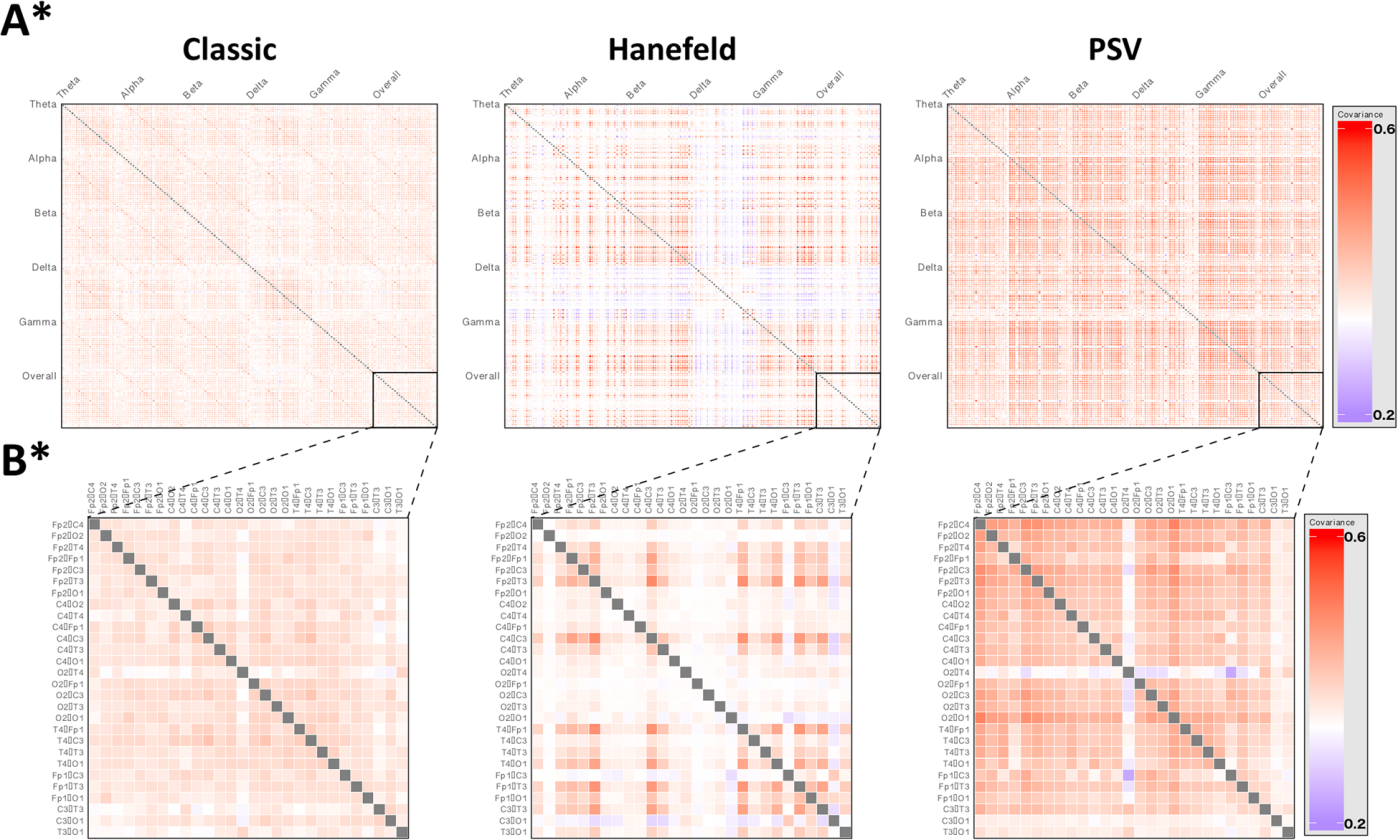
Clinical phenotypes show differences in network architecture. All subplots depicting a statistical comparison significant at a *p* value of < 0.05 are marked with an asterisk (*). **a** Covariance matrices of inter-electrode coherence measurements for Classic (n = 26), Hanefeld (n = 4) and PSV (n = 5) groups, allowing visualisation of higher-order network function. Each row and each column represent a pair of electrodes. These are arranged into blocks along the axes, with measures for each electrode pair at all frequency bands and across the overall spectrum. The intensity of each cell represents the covariance between activity in the corresponding electrode pairs at the corresponding frequency band. Visualisation suggests differences in network activities between phenotypes; comparison of first principal components indicated a statistically significant difference between groups (p < 0.0001, Kruskal-Wallis H test), indicating that there are differences in network architecture between clinical phenotypes. The Classic group demonstrates a pattern of low network involvement of left and right sided occipital areas. The Hanefeld network pattern is characterised by very low covariance across all pairs in delta band, including cross-frequency, indicating abnormalities in network function within this frequency range, as well as very low network involvement of right occipital and parietal regions. The PSV groups demonstrates a similar overall pattern to the Classic group, though with greater overall covariance between pairs and a more marked reduction in involvement of bilateral occipital regions. **b** Subplots of overall covariance matrices in A, showing only covariance between electrode pairs over the whole power spectrum. Each row and each column represents an electrode pair as labelled. Closer examination of covariance within the overall spectrum suggests that differences seen across the whole matrix are still evident within the overall spectrum alone

We evaluated higher-order network function for each group using covariance matrices of inter-electrode coherence measures (Fig. 4a).

We explored the patterns of network activity differentiating the groups and found statistically significant differences in coherence of electrode pairs in the overall power spectrum between phenotypes (using a threshold of *p* < 0.05, Kruskal-Wallis H test). The results of this are shown in Fig. 4.

Right temporo-parietal connectivity (T4-C4) was found to differ between clinical phenotypes (*p* = 0.04), consistent with the patterns observed in the covariance matrices, with the Classic phenotype (0.42 +/− 0.22) demonstrating the greatest coherence, followed by PSV (0.34 +/− 0.19) and then Hanefeld (0.21 +/− 0.05), suggesting that specific network-level dysfunctions may play a role in determining the Rett phenotype expressed (see Additional file 1: Table S11, for coherence measures between each electrode pair).

#### Characterising the PSV variant

Having determined that network level dysfunctions may differ across clinical phenotypes and hence play a role in mediating the clinical presentation of a given mutation, we compared the PSV variant (*n* = 5) directly to a group of Classic phenotype (*n* = 26) with mutations in same gene (MeCP2).

Notably, comparison of the inter-electrode coherence profiles of PSV and Classic groups demonstrated a statistically significant difference in left-sided parieto-occipital coherence (PSV: 0.55 +/− 0.07, Classic: 0.39 +/− 0.17; *p* = 0.026, Mann-Whitney U test), shown in Additional file 1: Figure S8. This suggests a role for parieto-occipital network function in mediating the phenotype of PSV variant Rett syndrome, with increased left-sided parieto-occipital connectivity potentially associated with a preservation of speech function.

### Longitudinal analysis

**Fig. 5:**
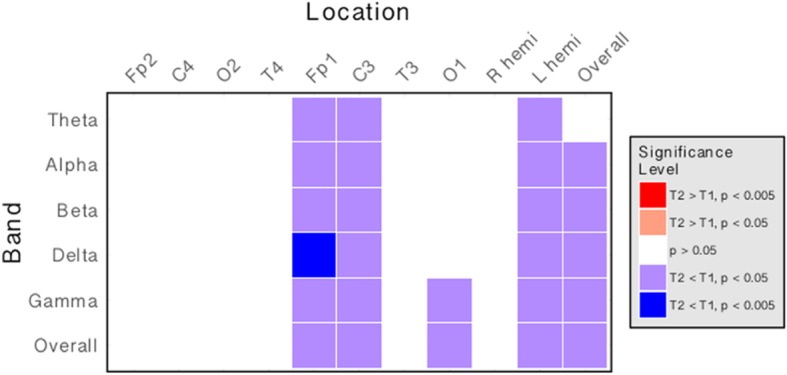
Spectral power is reduced across multiple bands at 10–14 months follow-up. Matrix illustrating the direction and magnitude of differences in spectral power at each frequency band and at each electrode location between timepoint 1 (0 months) and timepoint 2 (10–14 months). Each column represents a specific electrode location. Each row represents a frequency band. The colour of each cell indicates the direction of change (red: increase over time; blue: decrease over time). The intensity of the cell indicates the statistical threshold crossed (Wilcoxon signed rank test). There is a decrease in overall spectral power on follow-up, particularly in left frontal and parietal areas, indicating that spectral power profile changes with age

Comparison of spectral power profiles at baseline and at follow-up revealed changes in the power and distribution of frequency bands over time (Fig. 5). We found a generalised decrease in overall power across the scalp, and also in individual bands (*p* < 0.05, Wilcoxon signed rank test; see Additional file 1: Table S12, for comparisons of power at each electrode location at baseline and at follow-up). This pattern of decreasing power with age is particularly evident in left frontal (Fp1) and parietal (C3) regions. This suggests that spectral power profiles, repeated over ten-minute epochs at each sample point, are not entirely stable with time.

Notably, we found no statistically significant differences in comparisons of asymmetry (see Additional file 1: Table S13) or inter-electrode coherence profiles (see Additional file 1: Table S14) at follow up (*p* > 0.05, Wilcoxon signed rank test). This indicates that patterns of network activity are stable across time, and as a result network features that characterise specific subgroups of Rett Syndrome may have a role as valuable markers, as well as providing insights into the link between genetic mutation and clinical syndrome.

Notably, the absolute change in overall power measures was not significantly correlated with severity of disease as assessed by the International Severity Score (*r* = 0.62, *p* = 0.072), nor was the change in overall asymmetry (*r* = − 0.03, *p* = 0.94). These relationships remained true when the differences were normalised with respect to the follow-up interval to give a rate of change (power: *r* = 0.59, *p* = 0.09; asymmetry: r = − 0.03, *p* = 0.93).

### Electrophysiological characterisation of epilepsy status

Patients were divided into No Epilepsy (*n* = 18), Epilepsy (*n* = 16) or Resistant Epilepsy (*n* = 8) groups in order to investigate whether these subtypes are characterised by specific functional patterns which may act as electrophysiological biomarkers of epilepsy status.

#### Spectral power profile

**Fig. 6:**
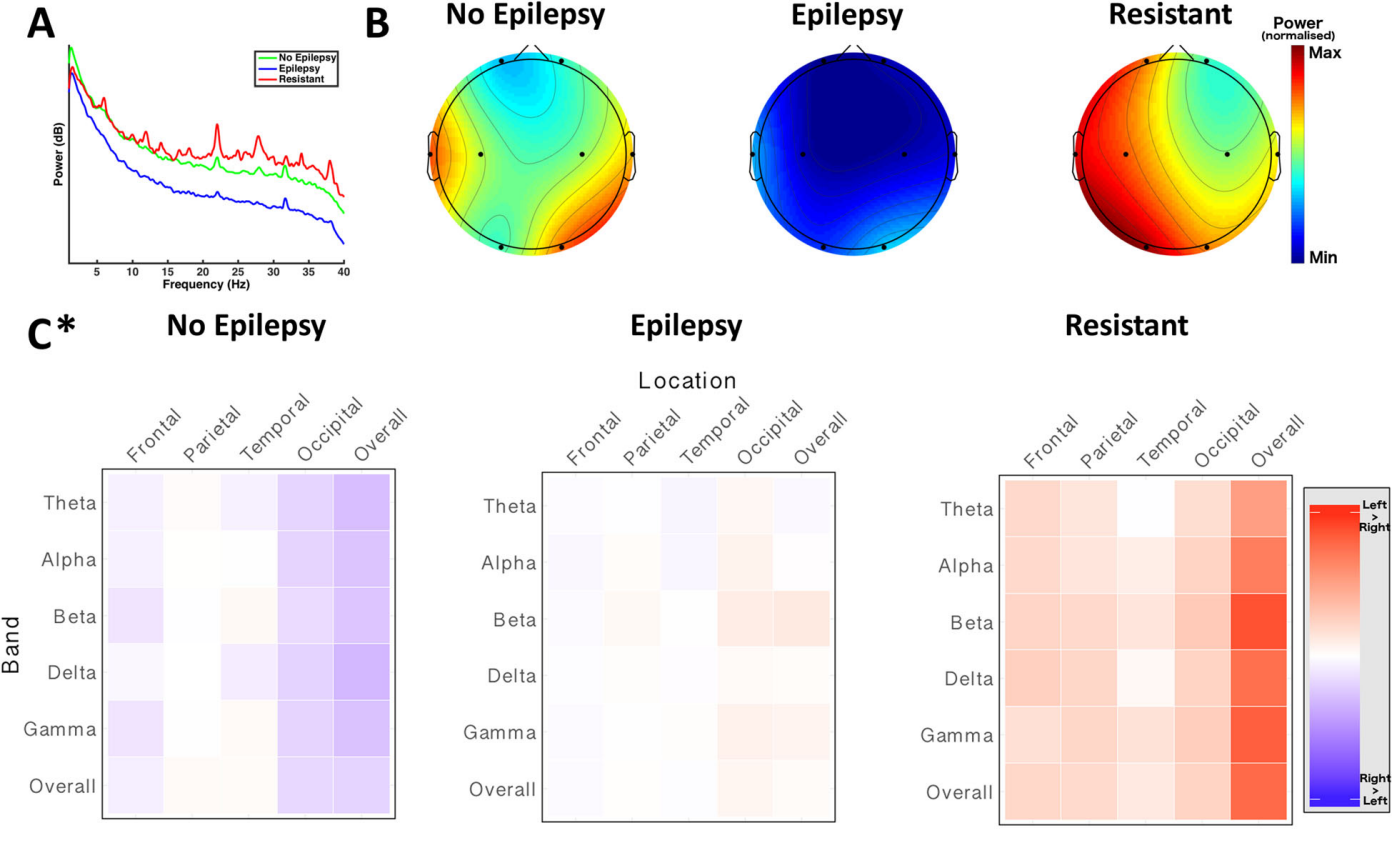
Spectral power profiles of epilepsy status groups. All subplots depicting a statistical comparison significant at a *p* value of < 0.05 are marked with an asterisk (*). **a** Overall power spectra between 1 Hz and 40 Hz for No Epilepsy (*n* = 18, green), Epilepsy (*n* = 16, blue) and Resistant (*n* = 8, red) groups. The Epilepsy group shows a tendency towards lower power throughout the power spectrum, though the differences in overall spectra between epilepsy status groups were not statistically significant (Kruskal-Wallis H test; p > 0.05). **b** Head plots demonstrate the spatial distribution of overall spectral power in No Epilepsy (*left*), Epilepsy (*middle*) and Resistant (*right*) groups. Dots represent electrode locations. Colour maps show relative power of the overall spectrum interpolated between electrodes. Colour maps were calculated using the maximum (red) and minimum (blue) across the entire population and applied to all groups, allowing direct comparison of power distribution between groups. The differences in power at each individual electrode were not statistically significant (Kruskal-Wallis H test p > 0.05). **c** Profile of hemispheric asymmetry at each frequency band by electrode location for No Epilepsy (*left*), Epilepsy (*middle*) and Resistant (*right*) groups. Each column represents a scalp location. Each row represents a frequency band. Cell colour is determined by the asymmetry of the corresponding band at the corresponding location, calculated by subtracting the power in that band at that location on one side from the other. Red indicates greater power in the left hemisphere, blue indicates greater power in the right hemisphere, and intensity indicates the magnitude of the asymmetry. The No Epilepsy group demonstrates a trend toward right hemispheric predominance in the overall spectrum, the Epilepsy group do not appear to demonstrate any hemispheric predominance, while the Resistant group show a tendency towards left hemispheric predominance in the overall spectrum; differences in the overall spectrum were statistically significant between groups (*p* < 0.05, Kruskal-Wallis H test)

The power spectra of the No Epilepsy, Epilepsy and Resistant epilepsy status groups between 1 Hz and 50 Hz were characterised (Fig. 6).

Although the Epilepsy group trends towards lower power across the spectrum, there was no statistically significant difference in overall power between the epilepsy status groups (No Epilepsy: 4.12 +/− 8.93 Epilepsy: − 0.24 +/− 9.22 Resistant: 5.69 +/− 8.78; *p* = 0.16, Kruskal-Wallis H test), indicating no gross difference in overall cortical electrical activity between groups (Fig. 6a).

The distribution of the overall power across the scalp was investigated (Fig. 6b). Although visualization suggests differences in distribution of power, quantitative assessments of these differences were not statistically significant (*P* > 0.05, Kruskal-Wallis H test; see Additional file 1: Table S15, for results of comparisons of overall spectrum at each electrode location).

The distribution of power within individual bands was investigated (Additional file 1: Figure S9). The distribution of all frequency bands is broadly similar within groups, with a similar pattern to that seen in the overall spectrum (see Additional file 1: Table S16, for results of comparisons of each frequency band).

#### Hemispheric asymmetry

Measures of hemispheric asymmetry were compared between epilepsy groups to evaluate whether there are differences in the balance of cortical functioning between groups (Fig. 6c).

Comparison of asymmetry in the overall power spectra demonstrated a statistically significant difference between groups in occipital regions (No Epilepsy: − 2.50 +/− 5.81 Epilepsy: 0.78 +/− 4.60 Resistant: 3.41 +/− 6.01; *p* = 0.04, Kruskal-Wallis H test), suggesting that differences in asymmetry across the full spectrum may differentiate between epilepsy status groups.

Further exploration of the hemispheric asymmetry profiles showed a pattern of statistically significant differences in asymmetry between groups (*p* < 0.05, Kruskal-Wallis H test; (see Additional file 1: Table S17, for comparisons of asymmetry at each electrode location)). The differences in occipital asymmetry seen in the overall spectrum were evident across all frequency bands (Additional file 1: Figure S10), indicating that patterns in the balance of activity in occipital areas is associated with epilepsy status.

The direction of differences in pairwise comparisons indicates that the Resistant group has the highest level of hemispheric asymmetry in occipital regions, while the Epilepsy group still has greater asymmetry than those in the No Epilepsy group. This suggests a pattern of increasing severity with increasing left-hemispheric predominance.

#### Network measures

**Fig. 7:**
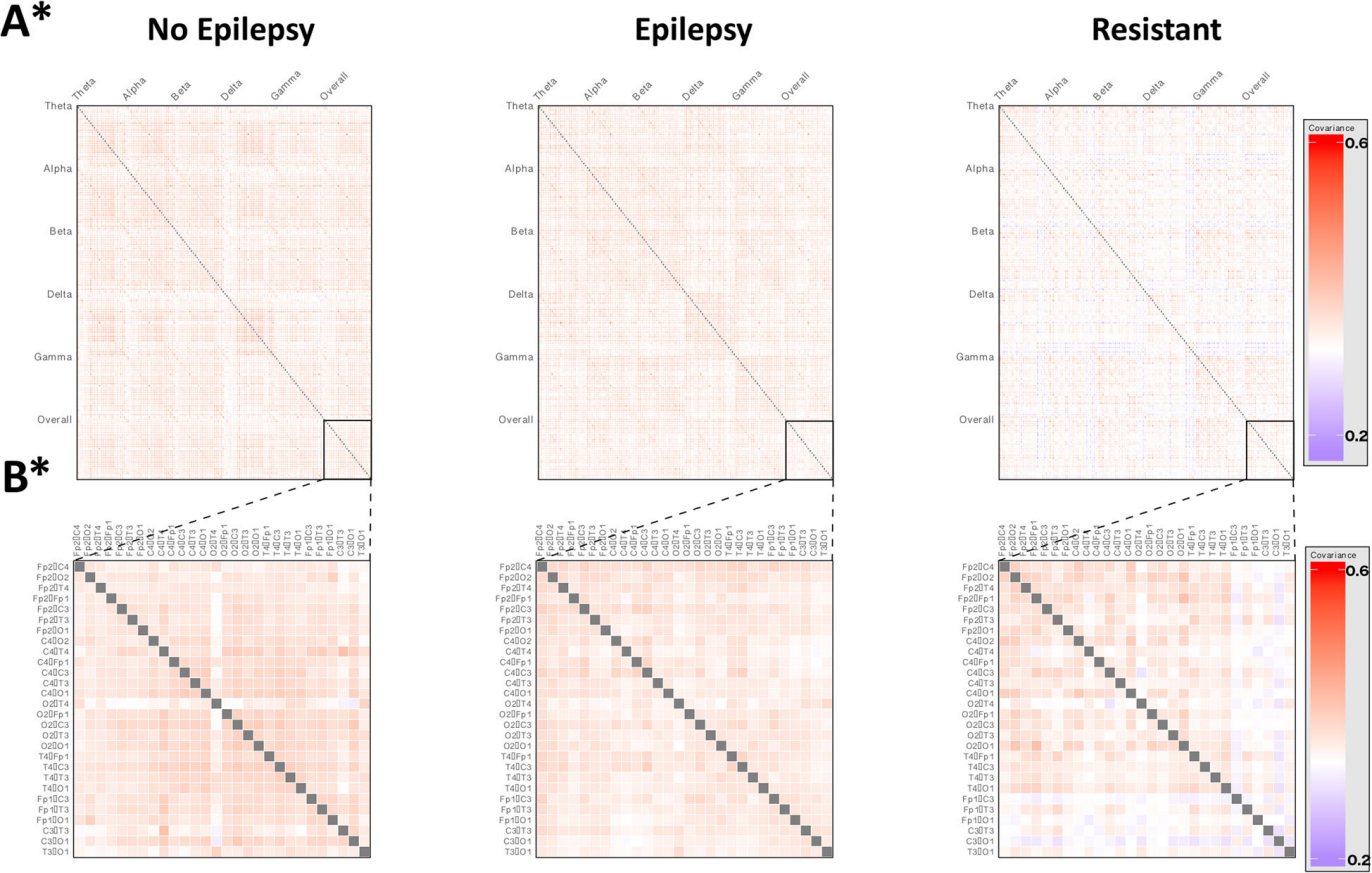
Epilepsy status groups are differentiated by electrophysiological measures. All subplots depicting a statistical comparison significant at a *p* value of < 0.05 are marked with an asterisk (*). **a** Covariance matrices of inter-electrode coherence measurements for No Epilepsy (n = 18, green), Epilepsy (*n* = 16, blue) and Resistant (n = 8, red) groups, allowing visualisation of higher-order network function. Each row and each column represent a pair of electrodes. These are arranged into blocks along the axes, with measures for each electrode pair at all frequency bands and across the overall spectrum. The intensity of each cell represents the covariance between activity in the corresponding electrode pairs at the corresponding frequency band. Visualisation suggests differences in network activities between epilepsy groups; comparison of first principal components indicated a statistically significant difference between groups (p < 0.05, Kruskal-Wallis H test), indicating that there are differences in network architecture between epilepsy groups. **b** Subplots of overall covariance matrices in A, showing only covariance between electrode pairs over the whole power spectrum. Each row and each column represents an electrode pair as labelled. Closer examination of covariance within the overall spectrum suggests that differences seen across the whole matrix are still evident within the overall spectrum alone

Profiles of network-level activity were compared between phenotypes in order to assess whether epilepsy status was associated with differences in patterns of network dysfunction (Fig. 7).

Comparison of first principal components of inter-electrode coherence measures across all frequency bands demonstrated a statistically significant difference in network architecture between groups (p = 0.04), Kruskal-Wallis H test), suggesting aberrations of network architecture with epilepsy status. A breakdown of the percentage of the total variance explained by each of the first five principal components for each group can be found in Additional file 1: Table S18.

Higher-order network function was visualised using covariance matrices of inter-electrode coherence measures (Fig. 7a).

The patterns of network activity differentiating the groups were explored. No individual electrode pairs were found to differ significantly between epilepsy status groups in the overall spectrum (*p* > 0.05, Kruskal-Wallis H test), indicating that despite the overall differences in network architecture, differences in the strength of individual electrode pairs between epilepsy status groups are not sufficiently large to reach statistical significance (see Additional file 1: Table S19, for coherence measures between each electrode pair).

These results indicate that there are differences in overall electrophysiological profile based on epilepsy status, and that these groups are primarily differentiated electrophysiologically by asymmetry of electrical activity in the occipital region in Rett Syndrome, with increasing severity associated with the presence of epilepsy and with treatment-resistance, as well as differences in overall network architecture.

#### Characterising epilepsy & treatment resistance

Having established that electrophysiological differences exist between epilepsy status groups, the Epilepsy and Resistant groups were combined into one overall group of patients with epilepsy, irrespective of treatment resistance, and compared to the No Epilepsy group in order to assess whether specific features drive the electrophysiologic profile evident in epilepsy in an effort to identify markers that may aid the prediction of epilepsy status in a patient whose epilepsy status is otherwise unknown.

Analysis of the features that had emerged as differing between groups demonstrated no difference in overall network architecture as assessed by principal component analysis of inter-electrode coherence measures (*p* = 0.970, Mann-Whitney U test), and a strong pattern of difference in overall occipital asymmetry (No Epilepsy: − 2.50 +/− 5.81, Epilepsy: 1.66 +/− 5.14; *p* = 0.019, Mann-Whitney U test), as well as differences in all individual frequency bands, indicating that asymmetry in the occipital regions is the primary driver of differences in electrophysiological profile evident in patients with epilepsy (see Additional file 1: Table S20, for results of all asymmetry comparisons).

The Epilepsy and Resistant groups were then directly compared in order to assess whether, in a population with epilepsy, treatment resistance was associated with specific abnormalities. Analysis of the features that had been identified on Kruskal-Wallis testing demonstrated strong differentiation based on overall network architecture (*p* = 0.029, Mann-Whitney U test), while differences in occipital asymmetry measures did not emerge as statistically significant (Epilepsy: 0.78 +/− 4.60, Resistant: 3.41 +/− 6.01; *p* = 0.444, Mann-Whitney U test) between groups, indicating that asymmetry measures differentiated less clearly between the treatment responsive and resistant groups (see Additional file 1: Table S21, for results of all asymmetry comparisons).

These results indicate that the presence of epilepsy is predominantly marked by differences in occipital asymmetry, while treatment resistance is indicated by network-level abnormalities.

## Discussion

We present the first quantitative electrophysiological characterization of Rett Syndrome variants, demonstrating that these variants are characterized by specific electrophysiological patterns, that these features are stable over time, and that these network-level abnormalities may contribute to the determination of epilepsy status and treatment responsiveness.

These findings provide insight into the specific abnormalities underlying different variants and presentations of RTT, and may have valuable applications in the clinical evaluation of RTT.

### Genetic and phenotypic groups are characterized by specific patterns of network activity

Both genetic and clinical variants of RTT were associated with distinct patterns of inter-electrode coherence measures, indicating differences in network architecture between these subgroups.

These results reveal a role for network dysfunction in mediating the clinical heterogeneity observed in RTT, providing a mechanism through which genetic abnormalities altering synaptic regulation produce varying clinical syndromes. This framework of RTT as a disorder of neural connectivity is consistent with the underlying molecular biology, with previous evidence of electrophysiological abnormalities, and with other related neurodevelopmental disorders such as ASD, where the role of aberrant network function is well established (Murias et al. [21]).

Notably, there were no statistically significant differences in measures of spectral power either in the overall spectrum or within specific bands both overall and between specific electrode locations, between genetic or clinical subgroups. This suggests that the observed differences are not simply due to a gross focal abnormality in neural functioning, but rather a more subtle, systemic alteration at the network level.

### Network architecture provides a stable endophenotype in RTT

Remarkably, longitudinal analysis of a subgroup of patients demonstrates that network measures are stable across time. This further supports a potential role for network-level dysfunction in mediating the phenotypic expression of specific genetic abnormalities, as the observed differences between subgroups appear to be conserved over time, rather than simply a transitory phenomenon at the time of testing.

This stable nature of the electrophysiologic characteristics of RTT variants suggests that they may prove useful as biomarkers for the classification and prognostication of RTT patients. Furthermore, greater characterisation of the electrophysiologic profiles of RTT subtypes may be able to provide an objective, electrophysiologic framework for the classification of this heterogeneous neurodevelopmental disorder, rather than the currently used clinical methods.

Interestingly, despite stability of network level features over time, we observed a widespread reduction in power at follow-up (Fig. 5). Although this analysis is limited by the relatively small numbers available for longitudinal analysis, the results suggest that the progression of RTT is marked by gradual reduction in global spectral power, a feature typically observed in advanced ageing and neurodegeneration, potentially offering insight into the neuropathological course of RTT. Greater characterization with a larger prospective cohort in a design more suited to evaluating such longitudinal changes may be warranted to further investigate the precise nature of these power abnormalities with disease progression.

### Occipito-temporal dysfunction drives differences in RTT subgroups

While the existence of stable neurophysiological correlates of genetic and phenotypic subtypes is interesting in itself, analysis of the nature of the between-groups differences offer insight into the nature of the dysfunctions responsible for the observed differences at the clinical level.

Notably, differences in global connectivity of occipital and temporal networks appear to be tightly linked to the clinical severity of RTT. Loss of these functioning within these networks is consistent with a loss of normal sensory integration, providing a logical neurologic correlate to the severity of neurodevelopmental deficit produced. Furthermore, preservation of speech in the PSV variant appears to be particularly related to increased integration of parietal networks, which may offer a mechanistic insight into the reasons for the language loss observed in the typical Rett phenotype.

Additionally, more clinically “severe” syndromes such as those produced by *CDKL5* mutations tend to be associated with marked abnormalities in within- and cross-frequency network function in delta band. Given typical associations of such slow-wave activity with unconsciousness, neurodegenerative conditions and temporal lobe epilepsy, abnormalities in delta network in these severe syndromes may play a role in the observed rapid regression, lack of responsiveness and treatment-refractory epilepsy.

### Epilepsy status is associated with specific network features in RTT

Both the presence of epilepsy and its response to treatment are also associated with specific electrophysiological features. Notably, the presence of epilepsy is largely driven by measures of occipital asymmetry across all bands. This is suggestive of an abnormal distribution of activity in occipital regions in patients with epilepsy, consistent with the proposed role of dysfunction in occipital networks in determining clinical severity. Given that epilepsy is classically attributed to abnormal synchronous neuronal discharges, this may suggest the presence of excessively active networks in the occipital regions acting as a focus for seizure onset in these patients, further underlining the particular role played by abnormalities of occipital network formation in the clinical phenotype of RTT.

Within those with clinically evident epilepsy, treatment resistance was marked by specific network-level abnormalities; in particular, a pattern of low global integration of frontotemporal networks was observed in those refractory to treatment, a pattern not observed as particularly characteristic of any genetic or clinical subtype. This suggests that reduced response to treatment is associated with increasingly disordered network architecture, beyond that observed in non-epileptic or treatment-responsive patients. This particularly disordered network function may increase seizure predisposition to such an extent that it is not overcome by medical therapies to the same extent as in those with less disordered architecture. Such an explanation also ties into the association of epilepsy with particular mutations and particularly severe clinical pictures, in that more “severe” network-level abnormalities may further increase the predisposition to epilepsy, and the likelihood that the epilepsy will be treatment-refractory.

Interestingly, as both coherence measures and asymmetry were demonstrated to remain stable over time, these features may serve as predictive markers of epilepsy and treatment responsiveness in RTT. This would provide a very valuable biomarker for the prediction of patient outcomes. A prospective analysis of the ability of occipital asymmetry and global measures of network integration to predict onset of epilepsy in new cases of RTT would be valuable in validating these features as clinically useful biomarkers in RTT.

Despite being the first report on the electrophysiological characterization of Rett Syndrome variants, the current study was limited by a relatively small number of the rare subtypes of RTT. This may have limited the ability to detect differences in some measures, particularly those marked by a large degree of variance. Several features showed interesting trends, such as a marked pattern of asymmetry observed in the *CDKL5* group, which may have reached statistical significance if greater statistical power were available, potentially offering even greater insight into the differences between these subtypes. However, for a disorder with a population prevalence of 1 per 10,000 live female births, and where the known variants are rare, our sample is very large and presents the first report on electrophysiological differences between these variants.

Additionally, the use of small numbers of electrodes limited the amount of electrophysiological data collected. Greater data availability may allow further characterisation of the specific features of each of these subtypes. This limitation was largely due to the need to use small caps with limited montages with the young children with behavioural disorders included in the study. Notably, marked differences at the network level were still distinguished despite these limitations, suggesting strong differences in these measures.

## Conclusion

This study represents the first electrophysiological characterisation of RTT subtypes. We demonstrate the role of dysfunctional network architecture as an important intermediary between genetic dysregulation of synapse formation and clinical phenotype. We further provide evidence for a specific role of occipito-temporal networks in the pathogenesis of RTT, and demonstrate that electrophysiological features are strongly associated with co-morbid epilepsy status. We show that network features appear to be stable over time, outlining potential value as a biomarker for diagnosis, classification and prognostication of RTT subtypes. Further work using a longitudinal analysis of a prospective cohort will allow greater elucidation of the prognostic value of electrophysiologic measures in this rare disorder.

## Additional file

Additional file 1: Supplementary information is included in one single file, named Supplementary Materials, and containing: Appendix 1–4 and **Tables S1–S21**; **Figures S1–S10** are included as separate files. (DOCX 596 kb)

## Abbreviations

CDLK5: Cyclin dependent kinase-like 5
EEG: Electroencephalogram
MeCP2: Methyl CpG binding protein 2
PSV: Preserved speech variant
RTT: Rett syndrome
