# Supplementary material for: Clinical and genetic Rett syndrome variants are defined by stable electrophysiological profiles
Source: BMC Pediatr. 2018 Oct 19;18:333. doi: 10.1186/s12887-018-1304-7 (PMC6195747; doi:10.1186/s12887-018-1304-7)
Supplement: Supplementary file 1 — Supplementary information is included in one single file, named Supplementary Materials, and containing: Appendix 1–4 and Tables S1–S21; Figures S1–S10 are included as separate files. (DOCX 596 kb) [file 12887_2018_1304_MOESM1_ESM.docx]

**Additional file 1**

**Appendix 1: Demographics**

All subjects were female. Handedness could not be meaningfully determined as the purposeful use of the hands was severely compromised in all patients. Further information on subject-level demographics are included in the following table. Note that some of those without clinical epilepsy were treated with antiepileptic medications as a prophylactic measure given the high incidence of epilepsy in this population.

**Table S1** Baseline Characteristics on Participants. NA = Data unavailable; PSV = Preserved Speech Variant

|  | **Age** | **Gene** | **Mutation** | **Presentation** | **Epilespy Status** | **Epilepsy Treatment** |
| --- | --- | --- | --- | --- | --- | --- |
| S1 | 4 | MECP2 | c. 763 C>T (p.R255X) | Classic | No |  |
| S2 | 4 | CDLK5 | Xp22.13 -153kb | Hanefeld | Resistant | NA |
| S3 | 1 | MECP2 | R106W (missense) | Classic | No |  |
| S4 | 3 | MECP2 | 7del46n | Classic | Yes | Valproate |
| S5 | 12 | MECP2 | Y141X (nonsense) | Classic | Yes | NA |
| S6 | 2 | MECP2 | P152R (missense) | NA | Yes | NA |
| S7 | 1 | CDLK5 | Del arr Xp22.13 | Hanefeld | Yes | Valproate |
| S8 | 8 | MECP2 | R168X (nonsense) | Classic | No |  |
| S9 | 4 | MECP2 | R270X (nonsense) | Classic | No | Valproate |
| S10 | 2 | MECP2 | C468G (missense) | Classic | No |  |
| S11 | 17 | MECP2 | R306H mosaic (missense) | Atypical Autism | Resistant | NA |
| S12 | 5 | MECP2 | Del ex4-3 | Classic | Yes | NA |
| S13 | 10 | MECP2 | R306C (missense) | PSV | Yes | Valproate |
| S14 | 8 | MECP2 | P152R (missense) | Classic | Resistant | Carbamezapine |
| S15 | 2 | MECP2 | 753delC (frameshift) | Classic | No |  |
| S16 | 12 | MECP2 | R168X (nonsense) | Classic | Yes | NA |
| S17 | 7 | MECP2 | R133C (missense) | PSV | Yes | NA |
| S18 | 11 | MECP2 | 1156 del 44 (frameshift) | PSV | Yes | NA |
| S19 | 7 | MECP2 | R168X (nonsense) | NA | Resistant | NA |
| S20 | 6 | MECP2 | R133C (missense) | Classic | No |  |
| S21 | 17 | MECP2 | Del ex3-4 | PSV | No |  |
| S22 | 6 | MECP2 | R133C (missense) | Classic | Yes | Valproate |
| S23 | 6 | MECP2 | T158M (missense) | Classic | Resistant | NA |
| S24 | 23 | MECP2 | 1096 del 89 (frameshift) | Classic | Resistant | NA |
| S25 | 3 | MECP2 | 1105 del 12 + 1157 del 44 | Classic | No |  |
| S26 | 11 | MECP2 | 1157 del 41 (frameshift) | Classic | No |  |
| S27 | 10 | MECP2 | NA | Classic | No |  |
| S28 | 6 | CDLK5 | Del 404-463/p.Asp135Phe154del20 | Hanefeld | Yes | NA |
| S29 | 2 | CDLK5 | NA | Hanefeld | Resistant | NA |
| S30 | 19 | MECP2 | Del ex3-4 | Atypical Autism | Yes | NA |
| S31 | 13 | MECP2 | P152R (missense) | Classic | Resistant | Valproate |
| S32 | 14 | MECP2 | T158M (missense) | PSV | Yes | NA |
| S33 | 8 | MECP2 | 1157 del 31 | Atypical Autism | Yes | NA |
| S34 | 6 | MECP2 | R168 (nonsense) | Classic | No |  |
| S35 | 6 | MECP2 | R294X (nonsense) | Classic | Yes | NA |
| S36 | 8 | MECP2 | T158M (missense) | Classic | No | Valproate |
| S37 | 3 | MECP2 | R270X (nonsense) | Classic | No |  |
| S38 | 5 | MECP2 | Ins ex 1 (frameshift) | Classic | No |  |
| S39 | 11 | MECP2 | 1162 del 26 (frameshift) | Classic | No |  |
| S40 | 5 | NA | Del 188 | NA | Yes | NA |
| S41 | 10 | MECP2 | R255X (nonsense) | Classic | Yes | NA |
| S42 | 3 | MECP2 | C916->T pR306C (frameshift) | NA | No |  |


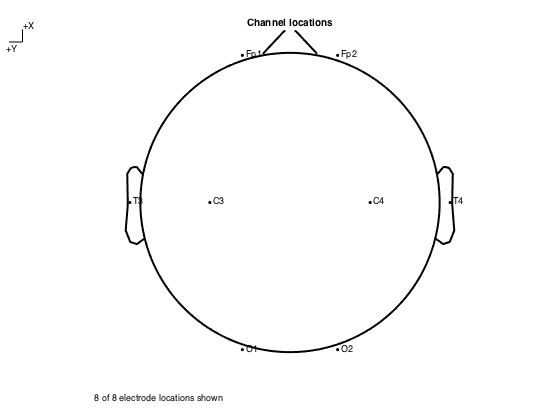


**Figure S1** Schematic of electrode locations. All recordings were performed using an eight-electrode montage, with bilateral temporal (Fp1 & Fp2), temporal (T3 & T4), parietal (C3 & C4) and occipital (O1 & O2) electrodes, positioned per the 10-20 system.

**Appendix 2: Epoch length**

In order to ensure that the results were not affected by artefacts or epileptic discharges despite the quality assurance steps taken, analyses were repeated using a series of shorter epochs which were then averaged.

The same ten minute segments were split into ten 60-second epochs and into sixty 10-second epochs. The spectra were then averaged across these epochs and all analyses repeated using these results.

These results showed no change in the nature of the results, indicating that the results derived from analysis of the ten minute epochs was not impacted by the presence of undetected artefacts in these longer epochs.

**Appendix 3: Subsampling Results**

A 15-fold subsampling method was employed on the larger MECP2 population, with subsamples repeatedly compared to the smaller CDKL5 population in order to ensure results are consistent.

*Spectral Power*

Comparison of spectral power between the 15 randomly drawn MECP2 subsamples and the CDKL5 population demonstrated no statistically significant differences across any of the 15 comparisons (p > 0.05, Mann-Whitney U test).

*Asymmetry*

Comparison of overall asymmetry measures and measures at each electrode location between the 15 MECP2 subsamples and the CDKL5 population demonstrated no statistically significant differences across any of the 15 comparisons (p > 0.05, Mann-Whitney U test).

*Network Measures*

Differences in the first principal components of inter-electrode coherence measures were statistically significant at the p < 0.05 level in 13 out of 15 comparisons (p = 0.0518 and 0.0551 in the two non-significant differences). Of these results, 9 of 15 were statistically significant following correction for multiple comparisons using the Bonferroni method (p < [0.05 / 15]). These results indicate that differences in overall network architecture between MECP2 and CDKL5 groups are consistently present across samples.

Comparisons of differences in inter-electrode coherence measures demonstrated statistically significant differences in a pattern consistent with that seen in the overall group, with differences predominantly in occipital and temporal pairs, in 10 of 15 comparisons at a p < 0.05 level. However, these results do not survive Bonferroni correction. The emergence of differences in occipital and temporal areas in the majority of comparisons is, however, consistent with the pattern observed across the overall population.

**Appendix 4: Severity correlations**

Overall power measures were not correlated with disease severity as assessed by the International Severity Score (r = 0.22, p = 0.14), nor was overall asymmetry (r = -0.07, p = 0.65).

*
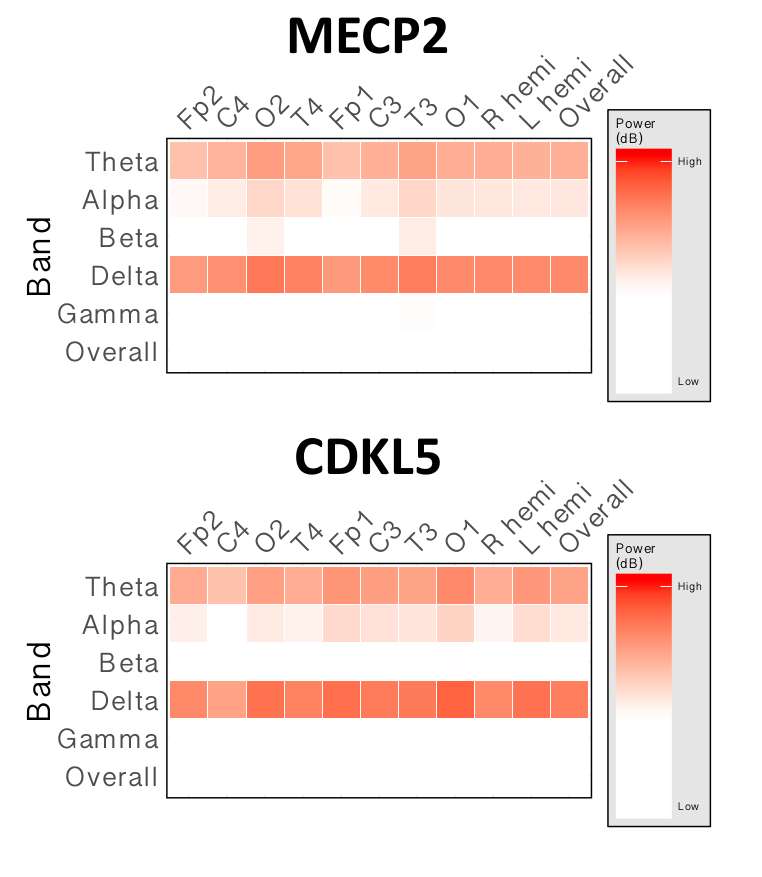
*

**Figure S2** Matrix of relative spectral power within each frequency band at each electrode location for MECP2 (*top*) and CDKL5 (*bottom*). Each column represents a specific electrode location. Each row represents a frequency band. The intensity of each cell represents the relative power at the corresponding location and frequencies, with white representing low power and red representing high power. Both groups were plotted using the same colour mapping, calculated based on maximum and minimum spectral power across the whole population, allowing direct comparison of intensities.

**
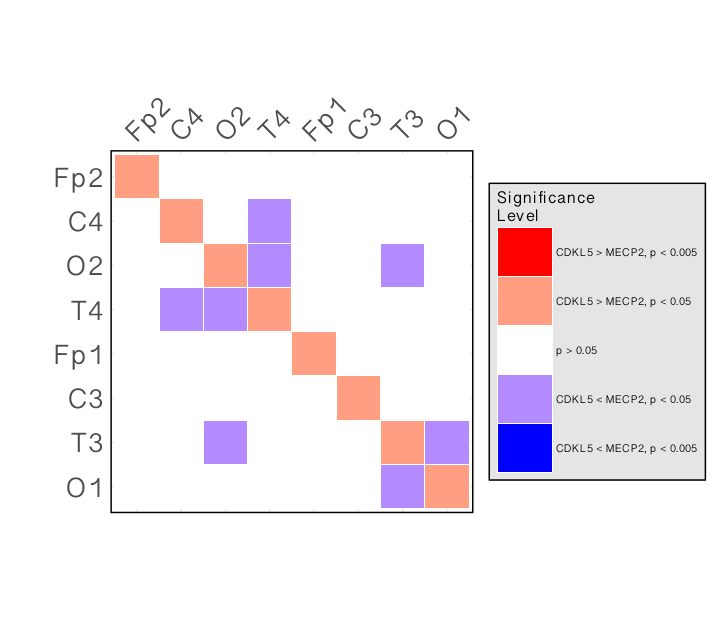
**

**Figure S3** Matrix illustrating the direction and magnitude of differences in inter-electrode coherence between groups. Each row and each column represent an electrode location, with each cell representing the coherence between the corresponding electrodes. Cell colour indicates the group with greater magnitude; here, a predominance of blue indicates greater power in the MECP2 group. Cell intensity indicates the statistical threshold crossed (Mann-Whitney U test). The major between-groups differences involve occipital and temporal electrodes, with greater coherence evident in the MECP2 group, suggesting dysfunctional occipito-temporal connectivity in the CDKL5 group.

**
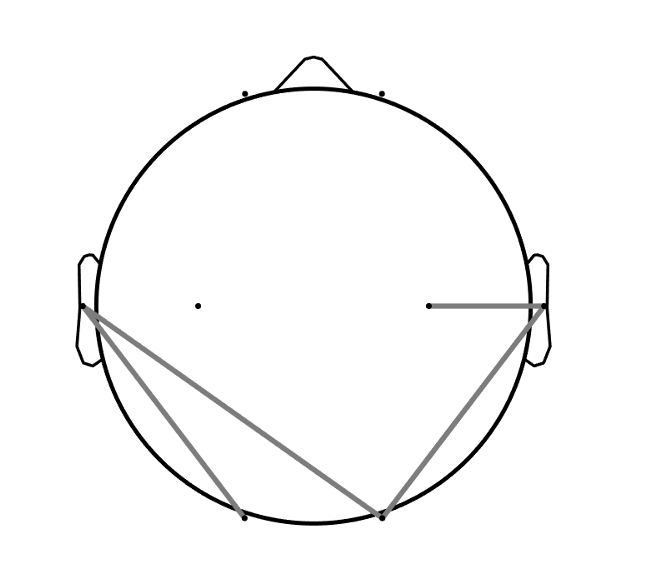
**

**Figure S4** Head plot demonstrating the spatial location of electrode pairs with significantly different coherence measurements between MECP2 and CDKL5 genetic subtypes (Mann-Whitney U test; p < 0.05). Lines represent an electrode pair with a coherence measurement found to differ significantly between groups. A bilateral pattern of differences in coherence in occipital and temporal regions appears to distinguish between groups, suggesting differences in occipito-temporal connectivity between genetic subtypes.

**Table S2** Overall power spectrum at each electrode location, MECP2 vs. CDKL5

|  | **MECP2** | **CDKL5** | **P** |
| --- | --- | --- | --- |
| **Fp2** | 0.78386 +/- 9.0676 | 1.2107 +/- 6.6491 | 0.769489566 |
| **C4** | 1.8061 +/- 10.0412 | -2.0554 +/- 7.9003 | 0.839232213 |
| **T4** | 4.4209 +/- 9.7445 | 1.1845 +/- 6.7475 | 0.839232213 |
| **O2** | 2.9339 +/- 9.4603 | 0.68492 +/- 7.6477 | 0.769489566 |
| **Fp1** | 0.4987 +/- 9.2586 | 4.0508 +/- 12.1209 | 0.513298347 |
| **C3** | 2.3166 +/- 9.7351 | 2.4735 +/- 13.7291 | 0.804166014 |
| **T3** | 4.8301 +/- 9.6344 | 2.2533 +/- 7.6801 | 0.735268153 |
| **O1** | 2.4117 +/- 9.1834 | 4.1707 +/- 12.6043 | 0.635944551 |
| **Right hemi** | 2.4862 +/- 8.8676 | 0.2563 +/- 7.1402 | 0.839232213 |
| **Left hemi** | 2.5143 +/- 8.7359 | 3.2371 +/- 10.9692 | 0.701564364 |
| **Overall** | 2.5002 +/- 8.5717 | 1.7466 +/- 8.6329 | 1 |

**Table S3** Power within each band at each electrode location, MECP2 vs. CDKL5

|  | **MECP2** | **CDKL5** | **p** |
| --- | --- | --- | --- |
| **Fp2 Theta** | 0.46981 +/- 26.3704 | 4.1618 +/- 4.9296 | 0.19883449 |
| **Fp2 Alpha** | 0.55312 +/- 21.8952 | 3.3243 +/- 3.52 | 0.169117124 |
| **Fp2 Beta** | 0.054983 +/- 18.1894 | 2.795 +/- 2.2749 | 0.130967749 |
| **Fp2 Delta** | 0.70159 +/- 30.347 | 4.6732 +/- 5.8704 | 0.232199468 |
| **Fp2 Gamma** | 0.50424 +/- 2.3696 | 1.123 +/- 0.75681 | 0.513298347 |
| **C4 Theta** | -0.25846 +/- 15.8254 | 13.2045 +/- 14.8306 | 0.061350003 |
| **C4 Alpha** | 0.30962 +/- 11.9428 | 9.9447 +/- 10.2085 | 0.061350003 |
| **C4 Beta** | 0.2551 +/- 7.8808 | 7.0191 +/- 6.0066 | 0.044833321 |
| **C4 Delta** | -0.94315 +/- 18.7344 | 15.8086 +/- 18.8213 | 0.061350003 |
| **C4 Gamma** | 1.1386 +/- 1.6758 | 1.9046 +/- 1.5323 | 0.183510003 |
| **T4 Theta** | -3.1252 +/- 31.6157 | 3.9303 +/- 4.2636 | 0.155569933 |
| **T4 Alpha** | -1.6942 +/- 23.5265 | 3.0063 +/- 3.1009 | 0.169117124 |
| **T4 Beta** | -1.339 +/- 17.1733 | 2.3633 +/- 1.727 | 0.142862771 |
| **T4 Delta** | -3.7071 +/- 39.621 | 4.8689 +/- 5.4879 | 0.155569933 |
| **T4 Gamma** | 1.1066 +/- 2.3588 | 1.3374 +/- 0.63062 | 0.169117124 |
| **O2 Theta** | -0.88496 +/- 8.2827 | 3.8539 +/- 3.8508 | 0.067868187 |
| **O2 Alpha** | -0.31914 +/- 6.0157 | 3.179 +/- 2.9569 | 0.067868187 |
| **O2 Beta** | 0.024796 +/- 3.9545 | 2.7014 +/- 2.0539 | 0.067868187 |
| **O2 Delta** | -1.5682 +/- 10.0877 | 4.6231 +/- 4.7531 | 0.074945533 |
| **O2 Gamma** | 1.1461 +/- 1.2384 | 1.4063 +/- 0.65701 | 0.355376274 |
| **Fp1 Theta** | -1.3982 +/- 9.9419 | 15.2909 +/- 27.73 | 0.169117124 |
| **Fp1 Alpha** | -0.65871 +/- 7.5738 | 11.1996 +/- 19.562 | 0.130967749 |
| **Fp1 Beta** | -0.40846 +/- 5.2609 | 7.7202 +/- 12.1503 | 0.082614409 |
| **Fp1 Delta** | -2.4895 +/- 13.1758 | 19.0681 +/- 35.1525 | 0.130967749 |
| **Fp1 Gamma** | 1.211 +/- 1.5211 | 2.4801 +/- 1.987 | 0.109495775 |
| **C3 Theta** | -28.0013 +/- 100.8649 | -5.6413 +/- 21.3361 | 1 |
| **C3 Alpha** | -21.4087 +/- 80.0088 | -3.2479 +/- 15.6834 | 1 |
| **C3 Beta** | -12.395 +/- 49.8421 | -0.84824 +/- 9.9367 | 0.982015816 |
| **C3 Delta** | -36.4843 +/- 131.5414 | -7.6136 +/- 26.4833 | 0.946083977 |
| **C3 Gamma** | 2.9247 +/- 8.854 | 0.89882 +/- 1.2621 | 0.804166014 |
| **T3 Theta** | 3.8273 +/- 9.9754 | 2.7771 +/- 3.2793 | 0.542771702 |
| **T3 Alpha** | 3.1859 +/- 8.1187 | 2.385 +/- 2.5137 | 0.604137334 |
| **T3 Beta** | 2.566 +/- 5.9956 | 2.1603 +/- 1.6374 | 0.542771702 |
| **T3 Delta** | 4.9135 +/- 13.6128 | 3.2555 +/- 4.1397 | 0.635944551 |
| **T3 Gamma** | 0.86818 +/- 0.59843 | 1.3855 +/- 0.53336 | 0.090907383 |
| **O1 Theta** | -2.4006 +/- 12.1845 | 2.9829 +/- 3.5297 | 0.45695062 |
| **O1 Alpha** | -1.5672 +/- 9.2527 | 2.435 +/- 2.6 | 0.430134842 |
| **O1 Beta** | -0.67987 +/- 6.4645 | 2.2237 +/- 1.6738 | 0.332398094 |
| **O1 Delta** | -3.6963 +/- 16.1682 | 3.5657 +/- 4.4796 | 0.379331935 |
| **O1 Gamma** | 1.1493 +/- 1.4655 | 1.3291 +/- 0.34869 | 0.430134842 |
| **R hemi Theta** | -0.94966 +/- 9.5096 | 6.2875 +/- 5.9061 | 0.067868187 |
| **R hemi Alpha** | -0.28764 +/- 6.9642 | 4.8636 +/- 4.2082 | 0.090907383 |
| **R hemi Beta** | -0.25102 +/- 5.3705 | 3.7197 +/- 2.5301 | 0.074945533 |
| **R hemi Delta** | -1.3792 +/- 11.4128 | 7.4935 +/- 7.4615 | 0.082614409 |
| **R hemi Gamma** | 0.97388 +/- 0.86156 | 1.4428 +/- 0.78217 | 0.119855566 |
| **L hemi Theta** | -6.9931 +/- 27.4316 | 3.8524 +/- 4.2388 | 0.142862771 |
| **L hemi Alpha** | -5.1122 +/- 21.5455 | 3.1929 +/- 3.2592 | 0.169117124 |
| **L hemi Beta** | -2.7294 +/- 13.4977 | 2.814 +/- 2.25 | 0.119855566 |
| **L hemi Delta** | -9.439 +/- 35.9322 | 4.5689 +/- 5.3517 | 0.155569933 |
| **L hemi Gamma** | 1.5383 +/- 2.5817 | 1.5234 +/- 0.40963 | 0.099857041 |
| **Overall Theta** | -3.9714 +/- 14.767 | 5.0701 +/- 4.9007 | 0.05535899 |
| **Overall Alpha** | -2.6999 +/- 11.3029 | 4.0283 +/- 3.5926 | 0.074945533 |
| **Overall Beta** | -1.4902 +/- 7.2742 | 3.2668 +/- 2.3038 | 0.040237894 |
| **Overall Delta** | -5.4092 +/- 19.3736 | 6.0311 +/- 6.2209 | 0.061350003 |
| **Overall Gamma** | 1.2561 +/- 1.4665 | 1.4831 +/- 0.59265 | 0.119855566 |

**Table S4** Hemispheric asymmetry, overall and within each band, MECP2 vs. CDKL5

|  | **MECP2** | **CDKL5** | **p** |
| --- | --- | --- | --- |
| **Frontal Theta** | -1.868 +/- 30.1703 | 11.1293 +/- 27.6823 | 0.668437759 |
| **Frontal Alpha** | -1.2118 +/- 24.5757 | 7.8752 +/- 19.4893 | 0.542771702 |
| **Frontal Beta** | -0.46345 +/- 19.8728 | 4.9252 +/- 12.3053 | 0.45695062 |
| **Frontal Delta** | -3.191 +/- 35.3402 | 14.3948 +/- 34.901 | 0.542771702 |
| **Frontal Gamma** | 0.7068 +/- 2.9032 | 1.3571 +/- 2.014 | 0.119855566 |
| **Frontal Overall** | -0.28512 +/- 5.813 | 2.8402 +/- 10.043 | 0.982015816 |
| **Parietal Theta** | -27.7427 +/- 104.6281 | -18.8456 +/- 34.5339 | 0.332398094 |
| **Parietal Alpha** | -21.7182 +/- 83.3698 | -13.1926 +/- 24.2431 | 0.332398094 |
| **Parietal Beta** | -12.6504 +/- 51.6264 | -7.8673 +/- 14.2545 | 0.310402256 |
| **Parietal Delta** | -35.5413 +/- 135.6599 | -23.422 +/- 43.4286 | 0.310402256 |
| **Parietal Gamma** | 1.7861 +/- 9.0484 | -1.0057 +/- 2.5985 | 0.769489566 |
| **Parietal Overall** | 0.5105 +/- 6.1854 | 4.529 +/- 8.9427 | 0.542771702 |
| **Temporal Theta** | 6.9525 +/- 32.7287 | -1.1532 +/- 2.3282 | 0.310402256 |
| **Temporal Alpha** | 4.88 +/- 24.2645 | -0.62124 +/- 1.6345 | 0.355376274 |
| **Temporal Beta** | 3.905 +/- 17.7885 | -0.20301 +/- 0.81703 | 0.430134842 |
| **Temporal Delta** | 8.6209 +/- 41.5691 | -1.6134 +/- 3.2362 | 0.289389508 |
| **Temporal Gamma** | -0.23838 +/- 2.5005 | 0.048043 +/- 0.14975 | 0.379331935 |
| **Temporal Overall** | 0.40914 +/- 6.9296 | 1.0688 +/- 2.8802 | 0.9102615 |
| **Occipital Theta** | -1.5156 +/- 13.9066 | -0.87108 +/- 3.1759 | 0.635944551 |
| **Occipital Alpha** | -1.248 +/- 10.3941 | -0.74407 +/- 2.2603 | 0.668437759 |
| **Occipital Beta** | -0.70463 +/- 6.8464 | -0.47774 +/- 1.5204 | 0.804166014 |
| **Occipital Delta** | -2.1281 +/- 17.5468 | -1.0573 +/- 3.8274 | 0.735268153 |
| **Occipital Gamma** | 0.0032627 +/- 1.7443 | -0.077168 +/- 0.53005 | 0.701564364 |
| **Occipital Overall** | -0.52219 +/- 5.5636 | 3.4858 +/- 8.3755 | 0.542771702 |
| **Overall Theta** | -24.1738 +/- 114.0943 | -9.7408 +/- 12.4182 | 0.513298347 |
| **Overall Alpha** | -19.298 +/- 90.7198 | -6.6828 +/- 8.9758 | 0.513298347 |
| **Overall Beta** | -9.9131 +/- 58.0204 | -3.6227 +/- 5.2117 | 0.701564364 |
| **Overall Delta** | -32.2398 +/- 146.5024 | -11.698 +/- 14.871 | 0.484680799 |
| **Overall Gamma** | 2.2578 +/- 9.9694 | 0.3222 +/- 1.5707 | 0.982015816 |
| **Overall Overall** | 0.11238 +/- 16.0031 | 11.9237 +/- 26.6892 | 0.604137334 |

**Table S5** Percentage of total variance explained by each of the first five principal components of the coherence measures for each genetic variant. P value represents value of statistical comparison of corresponding principal components using Mann-Whitney U test.

| **Principal Component** | **MECP** | **CDKL5** | **p** |
| --- | --- | --- | --- |
| 1 | 45.93 | 65.97 | 2.59x10^-8 |
| 2 | 8.96 | 23.21 | 0.52 |
| 3 | 7.39 | 10.82 | 1.12x10^-4 |
| 4 | 4.71 | 4.38x10^-14 | 0.54 |
| 5 | 4.38 | 2.68x10^-14 | 0.06 |

**Table S6** Inter-electrode coherence measures in the overall spectrum, MECP2 vs. CDKL5

|  | **MECP2** | **CDKL5** | **p** |
| --- | --- | --- | --- |
| **Fp2-C4** | 0.38826 +/- 0.17658 | 0.26799 +/- 0.099354 | 0.19883449 |
| **Fp2-O2** | 0.39432 +/- 0.18345 | 0.24101 +/- 0.044904 | 0.130967749 |
| **Fp2-T4** | 0.31762 +/- 0.14647 | 0.25148 +/- 0.12401 | 0.355376274 |
| **Fp2-Fp1** | 0.43861 +/- 0.18092 | 0.3857 +/- 0.19208 | 0.573064833 |
| **Fp2-C3** | 0.35785 +/- 0.16664 | 0.31233 +/- 0.13419 | 0.735268153 |
| **Fp2-T3** | 0.31678 +/- 0.13929 | 0.32992 +/- 0.24287 | 0.701564364 |
| **Fp2-O1** | 0.34673 +/- 0.14417 | 0.31559 +/- 0.062702 | 0.946083977 |
| **C4-O2** | 0.37778 +/- 0.1671 | 0.27348 +/- 0.072159 | 0.289389508 |
| **C4-T4** | **0.39859 +/- 0.21323** | **0.21085 +/- 0.049171** | **0.032236527** |
| **C4-Fp1** | 0.35279 +/- 0.17059 | 0.28079 +/- 0.077639 | 0.484680799 |
| **C4-C3** | 0.40986 +/- 0.1893 | 0.39591 +/- 0.25617 | 0.735268153 |
| **C4-T3** | 0.31367 +/- 0.1403 | 0.27058 +/- 0.13451 | 0.484680799 |
| **C4-O1** | 0.34374 +/- 0.16985 | 0.23366 +/- 0.067988 | 0.155569933 |
| **O2-T4** | **0.46452 +/- 0.22418** | **0.23521 +/- 0.081321** | **0.044833321** |
| **O2-Fp1** | 0.38081 +/- 0.15745 | 0.28758 +/- 0.053078 | 0.332398094 |
| **O2-C3** | 0.36951 +/- 0.17696 | 0.25152 +/- 0.035511 | 0.215050951 |
| **O2-T3** | **0.34202 +/- 0.14593** | **0.19227 +/- 0.052899** | **0.028775474** |
| **O2-O1** | 0.46784 +/- 0.18996 | 0.30112 +/- 0.16524 | 0.099857041 |
| **T4-Fp1** | 0.32023 +/- 0.15147 | 0.31489 +/- 0.23502 | 0.604137334 |
| **T4-C3** | 0.30856 +/- 0.17392 | 0.22987 +/- 0.068374 | 0.45695062 |
| **T4-T3** | 0.32974 +/- 0.15969 | 0.3129 +/- 0.11519 | 0.946083977 |
| **T4-O1** | 0.35083 +/- 0.15844 | 0.28326 +/- 0.19412 | 0.130967749 |
| **Fp1-C3** | 0.42255 +/- 0.18113 | 0.42625 +/- 0.13696 | 0.804166014 |
| **Fp1-T3** | 0.33559 +/- 0.14294 | 0.31097 +/- 0.23154 | 0.404255958 |
| **Fp1-O1** | 0.41215 +/- 0.16461 | 0.42887 +/- 0.17515 | 0.735268153 |
| **C3-T3** | 0.43542 +/- 0.19095 | 0.31247 +/- 0.20391 | 0.169117124 |
| **C3-O1** | 0.42482 +/- 0.16562 | 0.43952 +/- 0.22507 | 0.982015816 |
| **T3-O1** | **0.4582 +/- 0.16805** | **0.29736 +/- 0.11402** | **0.04986369** |

**
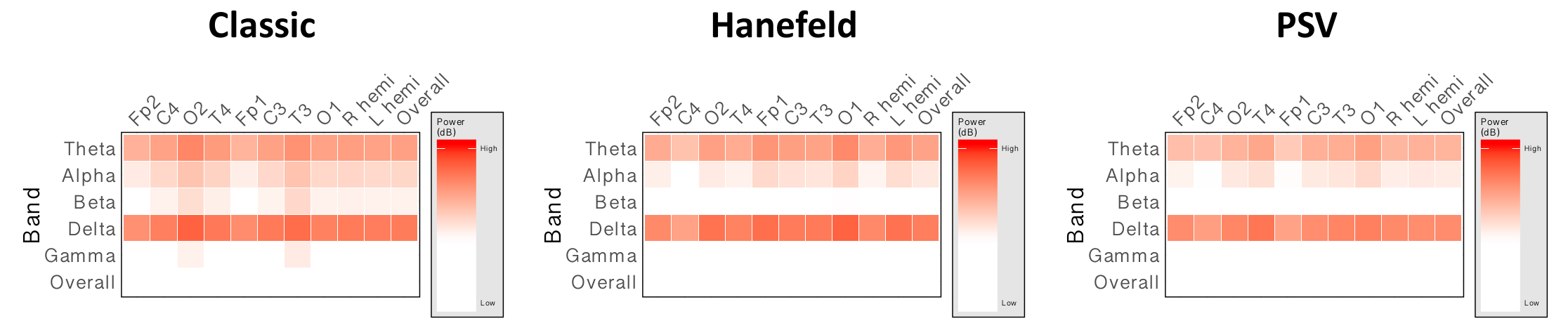
**

**Figure S5** Matrix of relative spectral power within each frequency band at each electrode location for Classic (*left*), Hanefeld (*middle*) and PSV (*right*) phenotypes. Each column represents a specific electrode location. Each row represents a frequency band. The intensity of each cell represents the relative power at the corresponding location and frequencies, with white representing low power and red representing high power. All groups were plotted using the same colour mapping, calculated based on maximum and minimum spectral power across the whole population, allowing direct comparison of intensities.

**
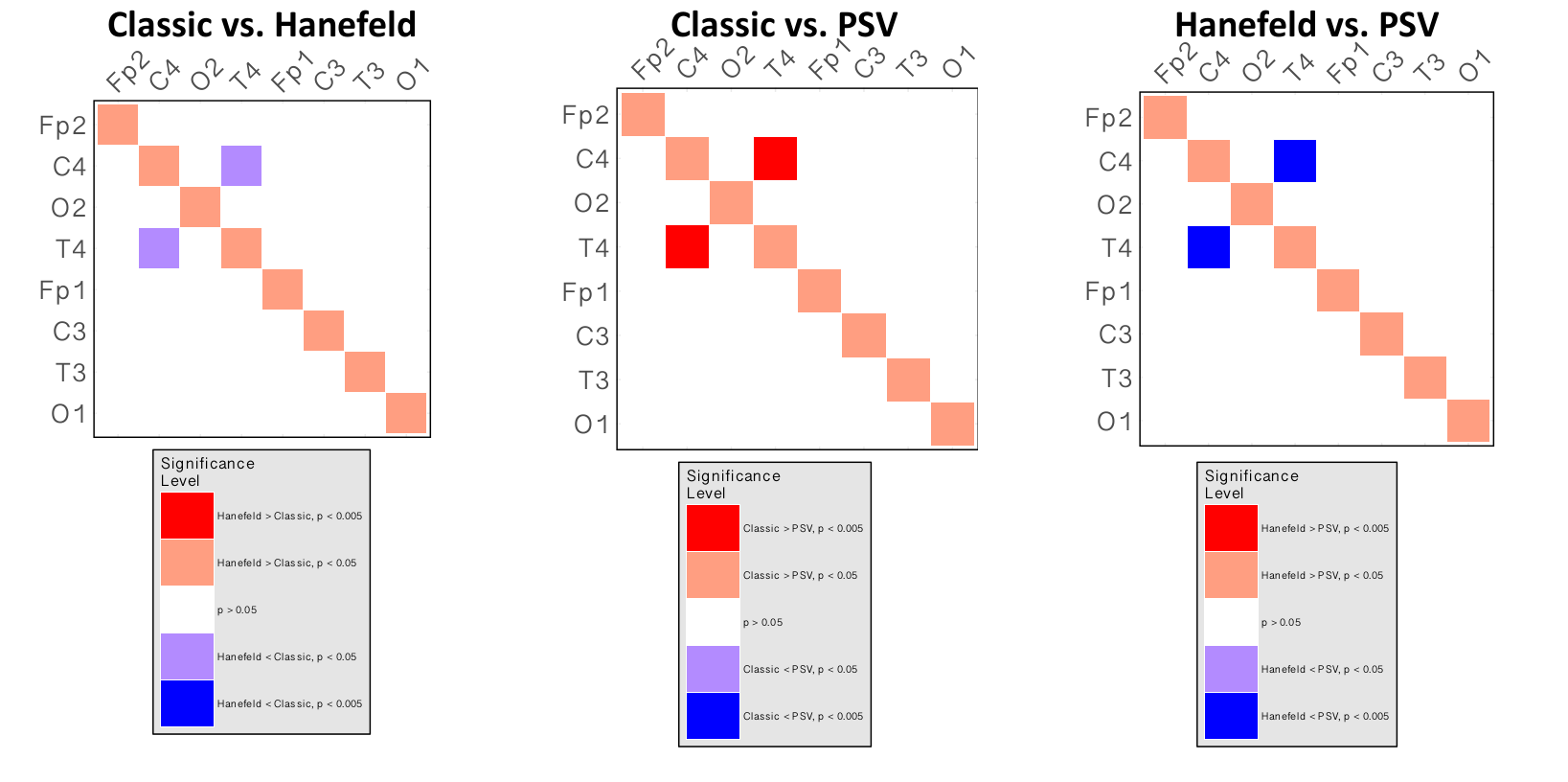
**

**Figure S6** Matrix illustrating the direction and magnitude of differences in inter-electrode coherence measures identified on Kruskal-Wallis testing in pairwise comparisons. Each row and each column represent an electrode location, with each cell representing the coherence between the corresponding electrodes. Note that whited-out cells were not identified as differing between phenotypic groups on Kruskal-Wallis testing and were therefore not included in pairwise comparisons. Of those compared, cell colour indicates the group with greater magnitude. Cell intensity indicates the statistical threshold crossed in pairwise comparison (Mann-Whitney U test). Comparisons between Classic and Hanefeld (*left*); Classic and PSV (*middle*); and Hanefeld and PSV (*right*) demonstrate greater right-sided temporo-parietal coherence in Classic than in both Hanefeld and PSV, and greater coherence in PSV than in Hanefeld, suggesting a potential role for temporo-parietal connectivity in the mediation of the language dysfunction common to Hanefeld and PSV patients.

**
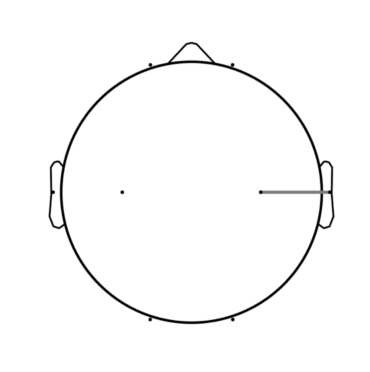
**

**Figure S7** Head plot demonstrating the spatial location of electrode pairs with significantly different coherence measurements between Classic, Hanefeld and PSV phenotypic subtypes (Kruskal-Wallis H test; p < 0.05). Lines represent an electrode pair with a coherence measurement found to differ significantly between groups. Right-sided temporo-parietal coherence differs between phenotypic groups, suggesting a role for these connections in determining the clinical phenotype of Rett syndrome.

**Table S7**  Overall power spectrum at each electrode location, Classic vs. Hanefeld vs. PSV

|  | **Classic** | **Hanefeld** | **PSV** | **p** |
| --- | --- | --- | --- | --- |
| **Fp2** | 1.997 +/- 9.2214 | 1.2107 +/- 6.6491 | 1.362 +/- 9.8788 | 0.892656783 |
| **C4** | 4.1835 +/- 10.4346 | -2.0554 +/- 7.9003 | -2.3331 +/- 3.4524 | 0.405922345 |
| **T4** | 6.4541 +/- 10.0642 | 1.1845 +/- 6.7475 | 1.3964 +/- 6.3454 | 0.393875026 |
| **O2** | 4.6804 +/- 9.6719 | 0.68492 +/- 7.6477 | 1.2297 +/- 7.7639 | 0.683873934 |
| **Fp1** | 1.8081 +/- 9.9568 | 4.0508 +/- 12.1209 | -0.75644 +/- 5.4416 | 0.845892803 |
| **C3** | 4.2752 +/- 9.6406 | 2.4735 +/- 13.7291 | 0.59003 +/- 6.1456 | 0.685993966 |
| **T3** | 6.6755 +/- 10.1421 | 2.2533 +/- 7.6801 | 2.4176 +/- 6.621 | 0.569568935 |
| **O1** | 4.073 +/- 9.5107 | 4.1707 +/- 12.6043 | 1.7815 +/- 2.9174 | 0.841843481 |
| **R hemi** | 4.3288 +/- 9.1627 | 0.2563 +/- 7.1402 | 0.41374 +/- 5.265 | 0.696795204 |
| **L hemi** | 4.208 +/- 8.9693 | 3.2371 +/- 10.9692 | 1.0082 +/- 4.5522 | 0.849868207 |
| **Overall** | 4.2684 +/- 8.8025 | 1.7466 +/- 8.6329 | 0.71097 +/- 4.4156 | 0.725683706 |

**Table S8** Power within each band at each electrode location, Classic vs. Hanefeld vs. PSV

|  | **Classic** | **Hanefeld** | **PSV** | **p** |
| --- | --- | --- | --- | --- |
| **Fp2 Theta** | 0.4389 +/- 30.9413 | 4.1618 +/- 4.9296 | -1.0647 +/- 3.9302 | 0.314256487 |
| **Fp2 Alpha** | 0.45531 +/- 25.6882 | 3.3243 +/- 3.52 | -0.66174 +/- 3.2345 | 0.265535344 |
| **Fp2 Beta** | -0.34105 +/- 21.3219 | 2.795 +/- 2.2749 | -0.027668 +/- 2.758 | 0.308581518 |
| **Fp2 Delta** | 0.72422 +/- 35.4841 | 4.6732 +/- 5.8704 | -1.5823 +/- 5.4068 | 0.410370048 |
| **Fp2 Gamma** | 0.14003 +/- 2.651 | 1.123 +/- 0.75681 | 1.4426 +/- 0.53926 | 0.133518333 |
| **C4 Theta** | -1.8909 +/- 16.9 | 13.2045 +/- 14.8306 | 8.2537 +/- 17.0658 | 0.177041055 |
| **C4 Alpha** | -0.87992 +/- 12.6458 | 9.9447 +/- 10.2085 | 6.7414 +/- 13.5703 | 0.177041055 |
| **C4 Beta** | -0.40669 +/- 8.6515 | 7.0191 +/- 6.0066 | 3.3757 +/- 7.5996 | 0.168601088 |
| **C4 Delta** | -2.8058 +/- 20.1521 | 15.8086 +/- 18.8213 | 8.4249 +/- 19.7441 | 0.183296842 |
| **C4 Gamma** | 1.1032 +/- 1.8934 | 1.9046 +/- 1.5323 | 1.2784 +/- 1.3795 | 0.141054682 |
| **T4 Theta** | -2.0872 +/- 35.7942 | 3.9303 +/- 4.2636 | -8.2506 +/- 25.4412 | 0.311306282 |
| **T4 Alpha** | -0.84872 +/- 26.6444 | 3.0063 +/- 3.1009 | -5.614 +/- 18.8539 | 0.345904205 |
| **T4 Beta** | -0.93227 +/- 19.7445 | 2.3633 +/- 1.727 | -2.8739 +/- 10.7487 | 0.323169361 |
| **T4 Delta** | -2.4358 +/- 45.2161 | 4.8689 +/- 5.4879 | -9.8789 +/- 28.9831 | 0.356647519 |
| **T4 Gamma** | 1.2529 +/- 2.7331 | 1.3374 +/- 0.63062 | 0.94564 +/- 0.68726 | 0.429667515 |
| **O2 Theta** | -1.8144 +/- 8.3698 | 3.8539 +/- 3.8508 | 3.8875 +/- 11.0445 | 0.151423612 |
| **O2 Alpha** | -1.0018 +/- 6.1023 | 3.179 +/- 2.9569 | 3.1756 +/- 7.8963 | 0.138071766 |
| **O2 Beta** | -0.40554 +/- 4.1252 | 2.7014 +/- 2.0539 | 1.9661 +/- 4.7663 | 0.149437188 |
| **O2 Delta** | -2.7501 +/- 10.2456 | 4.6231 +/- 4.7531 | 4.4092 +/- 13.073 | 0.130128771 |
| **O2 Gamma** | 1.208 +/- 1.3945 | 1.4063 +/- 0.65701 | 0.79919 +/- 0.95572 | 0.538328821 |
| **Fp1 Theta** | -1.4981 +/- 11.1542 | 15.2909 +/- 27.73 | -1.7763 +/- 8.8664 | 0.398446059 |
| **Fp1 Alpha** | -0.66408 +/- 8.427 | 11.1996 +/- 19.562 | -1.1968 +/- 7.2776 | 0.320293831 |
| **Fp1 Beta** | -0.43631 +/- 5.833 | 7.7202 +/- 12.1503 | -0.82416 +/- 5.1004 | 0.273566979 |
| **Fp1 Delta** | -2.712 +/- 14.8254 | 19.0681 +/- 35.1525 | -2.9314 +/- 11.4214 | 0.317990872 |
| **Fp1 Gamma** | 1.1681 +/- 1.6437 | 2.4801 +/- 1.987 | 1.7354 +/- 1.6739 | 0.34853058 |
| **C3 Theta** | -32.5467 +/- 114.0396 | -5.6413 +/- 21.3361 | -32.0452 +/- 81.3806 | 0.606064277 |
| **C3 Alpha** | -24.4731 +/- 90.0745 | -3.2479 +/- 15.6834 | -26.9154 +/- 68.0291 | 0.606064277 |
| **C3 Beta** | -15.4623 +/- 57.5835 | -0.84824 +/- 9.9367 | -9.2159 +/- 27.0914 | 0.581053707 |
| **C3 Delta** | -43.8433 +/- 150.5565 | -7.6136 +/- 26.4833 | -34.1147 +/- 88.0768 | 0.612088058 |
| **C3 Gamma** | 4.2187 +/- 9.8778 | 0.89882 +/- 1.2621 | -1.8898 +/- 5.6232 | 0.054980911 |
| **T3 Theta** | 4.8792 +/- 10.9916 | 2.7771 +/- 3.2793 | 5.0021 +/- 4.9227 | 0.469537399 |
| **T3 Alpha** | 4.0269 +/- 8.9365 | 2.385 +/- 2.5137 | 4.2088 +/- 3.8637 | 0.46941702 |
| **T3 Beta** | 3.261 +/- 6.567 | 2.1603 +/- 1.6374 | 3.1299 +/- 2.631 | 0.605842316 |
| **T3 Delta** | 6.3805 +/- 15.1948 | 3.2555 +/- 4.1397 | 5.9123 +/- 5.9058 | 0.546724902 |
| **T3 Gamma** | 0.95721 +/- 0.6104 | 1.3855 +/- 0.53336 | 0.61642 +/- 0.48865 | 0.063082302 |
| **O1 Theta** | -2.6068 +/- 12.8493 | 2.9829 +/- 3.5297 | -3.3174 +/- 15.9994 | 0.639639964 |
| **O1 Alpha** | -1.6163 +/- 9.6002 | 2.435 +/- 2.6 | -2.8613 +/- 12.8501 | 0.674816135 |
| **O1 Beta** | -0.79737 +/- 6.9112 | 2.2237 +/- 1.6738 | -1.1088 +/- 7.9835 | 0.653314939 |
| **O1 Delta** | -4.2712 +/- 17.5657 | 3.5657 +/- 4.4796 | -3.4667 +/- 18.4309 | 0.610006471 |
| **O1 Gamma** | 1.1809 +/- 1.665 | 1.3291 +/- 0.34869 | 1.0567 +/- 1.1688 | 0.717844841 |
| **R hemi Theta** | -1.3383 +/- 10.3116 | 6.2875 +/- 5.9061 | 0.7064 +/- 9.9249 | 0.198312326 |
| **R hemi Alpha** | -0.56874 +/- 7.5218 | 4.8636 +/- 4.2082 | 0.91021 +/- 7.3875 | 0.204269512 |
| **R hemi Beta** | -0.52139 +/- 5.9145 | 3.7197 +/- 2.5301 | 0.61003 +/- 4.6395 | 0.201497557 |
| **R hemi Delta** | -1.8169 +/- 12.4 | 7.4935 +/- 7.4615 | 0.34326 +/- 11.7154 | 0.219953161 |
| **R hemi Gamma** | 0.92602 +/- 0.93467 | 1.4428 +/- 0.78217 | 1.1165 +/- 0.75366 | 0.15861685 |
| **L hemi Theta** | -7.943 +/- 30.9844 | 3.8524 +/- 4.2388 | -8.0342 +/- 22.8837 | 0.375194262 |
| **L hemi Alpha** | -5.6817 +/- 24.2309 | 3.1929 +/- 3.2592 | -6.6913 +/- 18.8621 | 0.427391448 |
| **L hemi Beta** | -3.3588 +/- 15.5856 | 2.814 +/- 2.25 | -2.0047 +/- 7.8175 | 0.362030926 |
| **L hemi Delta** | -11.1113 +/- 41.1206 | 4.5689 +/- 5.3517 | -8.6501 +/- 24.9027 | 0.402435767 |
| **L hemi Gamma** | 1.8812 +/- 2.919 | 1.5234 +/- 0.40963 | 0.37971 +/- 1.4222 | 0.170782812 |
| **Overall Theta** | -4.6407 +/- 16.4354 | 5.0701 +/- 4.9007 | -3.6639 +/- 13.7517 | 0.174897863 |
| **Overall Alpha** | -3.1252 +/- 12.5307 | 4.0283 +/- 3.5926 | -2.8904 +/- 10.9095 | 0.208502927 |
| **Overall Beta** | -1.9401 +/- 8.263 | 3.2668 +/- 2.3038 | -0.69732 +/- 4.9706 | 0.143010795 |
| **Overall Delta** | -6.4641 +/- 21.9216 | 6.0311 +/- 6.2209 | -4.1536 +/- 15.0613 | 0.192320939 |
| **Overall Gamma** | 1.4036 +/- 1.6661 | 1.4831 +/- 0.59265 | 0.74809 +/- 0.82738 | 0.303837051 |

**Table S9** Hemispheric asymmetry, overall and within each band, Classic vs. Hanefeld vs. PSV

|  | **Classic** | **Hanefeld** | **PSV** | **p** |
| --- | --- | --- | --- | --- |
| **Frontal Theta** | -1.9369 +/- 35.4634 | 11.1293 +/- 27.6823 | -0.71171 +/- 7.8352 | 0.867084324 |
| **Frontal Alpha** | -1.1193 +/- 28.8867 | 7.8752 +/- 19.4893 | -0.53507 +/- 6.128 | 0.828186852 |
| **Frontal Beta** | -0.095248 +/- 23.368 | 4.9252 +/- 12.3053 | -0.7965 +/- 4.1316 | 0.781758983 |
| **Frontal Delta** | -3.4361 +/- 41.4692 | 14.3948 +/- 34.901 | -1.3491 +/- 9.7599 | 0.842506732 |
| **Frontal Gamma** | 1.0281 +/- 3.295 | 1.3571 +/- 2.014 | 0.29277 +/- 1.3359 | 0.356647519 |
| **Frontal Overall** | -0.18887 +/- 5.0656 | 2.8402 +/- 10.043 | -2.1185 +/- 11.0101 | 0.941471356 |
| **Parietal Theta** | -30.6555 +/- 117.5014 | -18.8456 +/- 34.5339 | -40.2997 +/- 91.0108 | 0.639944627 |
| **Parietal Alpha** | -23.5931 +/- 93.1473 | -13.1926 +/- 24.2431 | -33.6563 +/- 76.1595 | 0.639944627 |
| **Parietal Beta** | -15.056 +/- 59.5219 | -7.8673 +/- 14.2545 | -12.5914 +/- 30.7698 | 0.574756088 |
| **Parietal Delta** | -41.0374 +/- 154.6511 | -23.422 +/- 43.4286 | -42.5408 +/- 98.5343 | 0.609570907 |
| **Parietal Gamma** | 3.1156 +/- 10.0449 | -1.0057 +/- 2.5985 | -3.1681 +/- 6.2255 | 0.281047729 |
| **Parietal Overall** | 0.091656 +/- 6.8084 | 4.529 +/- 8.9427 | 2.9231 +/- 5.0777 | 0.70614802 |
| **Temporal Theta** | 6.9664 +/- 37.1937 | -1.1532 +/- 2.3282 | 13.2529 +/- 24.8801 | 0.324135576 |
| **Temporal Alpha** | 4.8754 +/- 27.5661 | -0.62124 +/- 1.6345 | 9.8228 +/- 18.4385 | 0.303837051 |
| **Temporal Beta** | 4.1933 +/- 20.5389 | -0.20301 +/- 0.81703 | 6.0037 +/- 10.342 | 0.316416476 |
| **Temporal Delta** | 8.8167 +/- 47.5816 | -1.6134 +/- 3.2362 | 15.7912 +/- 28.4893 | 0.293499565 |
| **Temporal Gamma** | -0.29566 +/- 2.9225 | 0.048043 +/- 0.14975 | -0.32921 +/- 0.98223 | 0.550291197 |
| **Temporal Overall** | 0.22139 +/- 7.5826 | 1.0688 +/- 2.8802 | 1.0212 +/- 7.4101 | 0.973757084 |
| **Occipital Theta** | -0.79234 +/- 14.4992 | -0.87108 +/- 3.1759 | -7.2048 +/- 18.1351 | 0.752069349 |
| **Occipital Alpha** | -0.61451 +/- 10.6519 | -0.74407 +/- 2.2603 | -6.037 +/- 14.2034 | 0.772946234 |
| **Occipital Beta** | -0.39179 +/- 7.2634 | -0.47774 +/- 1.5204 | -3.0749 +/- 8.4858 | 0.880607862 |
| **Occipital Delta** | -1.521 +/- 18.6555 | -1.0573 +/- 3.8274 | -7.876 +/- 21.577 | 0.809753304 |
| **Occipital Gamma** | -0.02713 +/- 1.9704 | -0.077168 +/- 0.53005 | 0.25756 +/- 1.489 | 0.884228034 |
| **Occipital Overall** | -0.60753 +/- 5.6986 | 3.4858 +/- 8.3755 | 0.55182 +/- 7.8694 | 0.750102221 |
| **Overall Theta** | -26.4186 +/- 129.7525 | -9.7408 +/- 12.4182 | -34.9624 +/- 88.3519 | 0.663322153 |
| **Overall Alpha** | -20.4514 +/- 102.71 | -6.6828 +/- 8.9758 | -30.4064 +/- 74.2557 | 0.635843893 |
| **Overall Beta** | -11.3494 +/- 67.2508 | -3.6227 +/- 5.2117 | -10.4588 +/- 32.6074 | 0.727666756 |
| **Overall Delta** | -37.1782 +/- 168.1499 | -11.698 +/- 14.871 | -35.9745 +/- 98.5882 | 0.66095737 |
| **Overall Gamma** | 3.8208 +/- 11.0882 | 0.3222 +/- 1.5707 | -2.947 +/- 6.2519 | 0.445502191 |
| **Overall Overall** | -0.48333 +/- 17.3764 | 11.9237 +/- 26.6892 | 2.3778 +/- 17.3876 | 0.874084038 |

**Table S10** Percentage of total variance explained by each of the first five principal components of the coherence measures for each phenotypic variant. P value represents value of statistical comparison of corresponding principal components using Kruskal-Wallis H test.

| **Principal Component** | **Classic** | **Hanefeld** | **Resistant** | **p** |
| --- | --- | --- | --- | --- |
| 1 | 43.05 | 65.97 | 71.91 | 5.36x10^-8 |
| 2 | 10.29 | 23.21 | 12.83 | 0.56 |
| 3 | 9.22 | 10.82 | 9.95 | 1.66x10^-5 |
| 4 | 6.34 | 4.38x10^-14 | 5.32 | 0.27 |
| 5 | 5.18 | 2.68x10^-14 | 7.47x10^-14 | 0.39 |

**Table S11** Inter-electrode coherence measures in the overall spectrum, Classic vs. Hanefeld vs. PSV

|  | **Classic** | **Hanefeld** | **PSV** | **p** |
| --- | --- | --- | --- | --- |
| **Fp2-C4** | 0.36939 +/- 0.14998 | 0.26799 +/- 0.099354 | 0.418 +/- 0.25513 | 0.419758 |
| **Fp2-O2** | 0.39957 +/- 0.18107 | 0.24101 +/- 0.044904 | 0.37598 +/- 0.20323 | 0.250663296 |
| **Fp2-T4** | 0.32081 +/- 0.13599 | 0.25148 +/- 0.12401 | 0.33025 +/- 0.20924 | 0.612592734 |
| **Fp2-Fp1** | 0.43339 +/- 0.1775 | 0.3857 +/- 0.19208 | 0.48946 +/- 0.20491 | 0.63683453 |
| **Fp2-C3** | 0.34698 +/- 0.15617 | 0.31233 +/- 0.13419 | 0.44253 +/- 0.24101 | 0.681548217 |
| **Fp2-T3** | 0.31837 +/- 0.13558 | 0.32992 +/- 0.24287 | 0.34503 +/- 0.21756 | 0.925910203 |
| **Fp2-O1** | 0.3349 +/- 0.13275 | 0.31559 +/- 0.062702 | 0.40578 +/- 0.18605 | 0.724807036 |
| **C4-O2** | 0.36999 +/- 0.1631 | 0.27348 +/- 0.072159 | 0.37665 +/- 0.17778 | 0.583132609 |
| **C4-T4** | **0.42425 +/- 0.22134** | **0.21085 +/- 0.049171** | **0.33674 +/- 0.18581** | **0.04471055** |
| **C4-Fp1** | 0.34744 +/- 0.17664 | 0.28079 +/- 0.077639 | 0.4063 +/- 0.15402 | 0.54085878 |
| **C4-C3** | 0.38498 +/- 0.18947 | 0.39591 +/- 0.25617 | 0.51336 +/- 0.19607 | 0.441352198 |
| **C4-T3** | 0.31258 +/- 0.14562 | 0.27058 +/- 0.13451 | 0.37665 +/- 0.16179 | 0.493611096 |
| **C4-O1** | 0.32268 +/- 0.16064 | 0.23366 +/- 0.067988 | 0.41441 +/- 0.17552 | 0.176493918 |
| **O2-T4** | 0.48098 +/- 0.22221 | 0.23521 +/- 0.081321 | 0.47967 +/- 0.29548 | 0.104367687 |
| **O2-Fp1** | 0.37058 +/- 0.15478 | 0.28758 +/- 0.053078 | 0.38415 +/- 0.16668 | 0.639944627 |
| **O2-C3** | 0.3546 +/- 0.17991 | 0.25152 +/- 0.035511 | 0.3904 +/- 0.21063 | 0.562426677 |
| **O2-T3** | 0.34777 +/- 0.14771 | 0.19227 +/- 0.052899 | 0.33654 +/- 0.17933 | 0.089920634 |
| **O2-O1** | 0.46419 +/- 0.18964 | 0.30112 +/- 0.16524 | 0.41621 +/- 0.24724 | 0.261112952 |
| **T4-Fp1** | 0.32378 +/- 0.15261 | 0.31489 +/- 0.23502 | 0.32021 +/- 0.1935 | 0.762527111 |
| **T4-C3** | 0.30527 +/- 0.18747 | 0.22987 +/- 0.068374 | 0.32699 +/- 0.19581 | 0.790990861 |
| **T4-T3** | 0.34025 +/- 0.16443 | 0.3129 +/- 0.11519 | 0.30814 +/- 0.20505 | 0.87393997 |
| **T4-O1** | 0.34039 +/- 0.1564 | 0.28326 +/- 0.19412 | 0.3788 +/- 0.19822 | 0.332371543 |
| **Fp1-C3** | 0.40177 +/- 0.15648 | 0.42625 +/- 0.13696 | 0.48468 +/- 0.23259 | 0.738758738 |
| **Fp1-T3** | 0.33425 +/- 0.14296 | 0.31097 +/- 0.23154 | 0.36687 +/- 0.17309 | 0.610990424 |
| **Fp1-O1** | 0.41033 +/- 0.16581 | 0.42887 +/- 0.17515 | 0.40638 +/- 0.12535 | 0.906363338 |
| **C3-T3** | 0.45337 +/- 0.19981 | 0.31247 +/- 0.20391 | 0.45669 +/- 0.19109 | 0.346189408 |
| **C3-O1** | 0.38811 +/- 0.16898 | 0.43952 +/- 0.22507 | 0.54912 +/- 0.074103 | 0.078213343 |
| **T3-O1** | 0.47543 +/- 0.17538 | 0.29736 +/- 0.11402 | 0.49078 +/- 0.096569 | 0.06862974 |

**
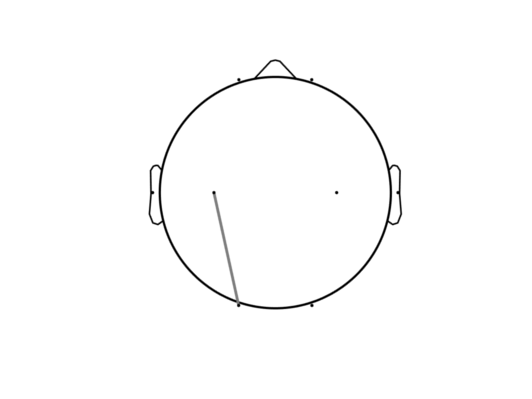
**

**Figure S8** Head plot demonstrating the spatial location of electrode pairs with significantly different coherence measurements between PSV (n = 5) and Classic (n = 26) subtypes (Mann-Whitney U test; p = 0.026). Lines represent an electrode pair with a coherence measurement found to differ significantly between groups. Left-sided parieto-occipital coherence differs between phenotypic groups, suggesting a role for these networks in mediating differences in language function in MECP2 variant Rett.

**Table S12** Overall power spectrum at each electrode location, baseline & at 10-14 months follow-up

|  | **T1** | **T2** | **p** |
| --- | --- | --- | --- |
| **Fp2 Theta** | -7.3729 +/- 38.81 | -31.4103 +/- 88.9653 | 0.00390625 |
| **Fp2 Alpha** | -6.3505 +/- 32.9227 | -21.9642 +/- 62.1145 | 0.00390625 |
| **Fp2 Beta** | -6.9692 +/- 29.9399 | -17.6147 +/- 50.9193 | 0.00390625 |
| **Fp2 Delta** | -7.5792 +/- 41.5921 | -41.9658 +/- 118.2243 | 0.0078125 |
| **Fp2 Gamma** | -0.23829 +/- 1.5369 | 0.52035 +/- 1.9936 | 0.07421875 |
| **Fp2 Overall** | 2.9917 +/- 7.4427 | -1.369 +/- 5.8999 | 0.42578125 |
| **C4 Theta** | 9.7998 +/- 10.7769 | -7.496 +/- 52.236 | 0.30078125 |
| **C4 Alpha** | 7.9414 +/- 7.9837 | -6.0164 +/- 39.0276 | 0.30078125 |
| **C4 Beta** | 5.5934 +/- 5.1042 | -9.1684 +/- 43.4681 | 0.42578125 |
| **C4 Delta** | 11.4973 +/- 13.2607 | -15.8196 +/- 83.8063 | 0.359375 |
| **C4 Gamma** | 0.84 +/- 1.3478 | -1.2499 +/- 7.7571 | 1 |
| **C4 Overall** | 4.5071 +/- 8.595 | -0.039413 +/- 5.4016 | 0.25 |
| **T4 Theta** | 3.9861 +/- 3.4114 | 7.1965 +/- 12.3109 | 0.65234375 |
| **T4 Alpha** | 3.2827 +/- 2.4413 | 5.8631 +/- 9.9845 | 0.734375 |
| **T4 Beta** | 2.3565 +/- 1.8198 | 6.0576 +/- 9.4184 | 0.42578125 |
| **T4 Delta** | 4.76 +/- 4.2228 | 8.9798 +/- 14.8037 | 0.65234375 |
| **T4 Gamma** | 0.89097 +/- 0.35707 | 2.1602 +/- 1.4948 | 0.0078125 |
| **T4 Overall** | 7.085 +/- 7.5867 | 1.0829 +/- 5.3506 | 0.09765625 |
| **O2 Theta** | 2.953 +/- 4.9703 | -3.8511 +/- 21.0348 | 0.25 |
| **O2 Alpha** | 2.4199 +/- 3.9833 | -3.4565 +/- 17.2391 | 0.25 |
| **O2 Beta** | 1.7031 +/- 3.2349 | -3.0579 +/- 15.867 | 0.42578125 |
| **O2 Delta** | 3.363 +/- 6.1926 | -3.7853 +/- 23.5562 | 0.25 |
| **O2 Gamma** | 0.57931 +/- 1.0389 | 0.36109 +/- 2.5081 | 0.42578125 |
| **O2 Overall** | 4.2003 +/- 6.6388 | -1.1398 +/- 6.4444 | 0.09765625 |
| **Fp1 Theta** | 13.2041 +/- 18.5791 | -2.1438 +/- 6.9222 | 0.01171875 |
| **Fp1 Alpha** | 10.162 +/- 13.387 | -1.4119 +/- 5.3839 | 0.01171875 |
| **Fp1 Beta** | 6.6905 +/- 8.2204 | -0.74812 +/- 3.9217 | 0.01953125 |
| **Fp1 Delta** | 16.6051 +/- 23.7113 | -2.9072 +/- 9.0526 | 0.01953125 |
| **Fp1 Gamma** | 0.60641 +/- 2.6078 | 1.2205 +/- 0.41009 | 0.359375 |
| **Fp1 Overall** | 5.4024 +/- 8.4499 | -2.9989 +/- 4.6568 | 0.0078125 |
| **C3 Theta** | -2.2297 +/- 13.2644 | 1.5216 +/- 8.9605 | 0.42578125 |
| **C3 Alpha** | -1.2014 +/- 9.5263 | 1.3813 +/- 6.4553 | 0.42578125 |
| **C3 Beta** | -0.32718 +/- 5.7336 | 1.3511 +/- 6.2534 | 0.42578125 |
| **C3 Delta** | -3.1592 +/- 16.6815 | 0.90089 +/- 11.3951 | 0.42578125 |
| **C3 Gamma** | 0.56888 +/- 0.59233 | 1.4509 +/- 1.635 | 0.09765625 |
| **C3 Overall** | 5.3502 +/- 8.8938 | -1.004 +/- 5.7636 | 0.0078125 |
| **T3 Theta** | 1.7766 +/- 3.3338 | 3.557 +/- 6.0989 | 0.359375 |
| **T3 Alpha** | 1.5677 +/- 2.7471 | 2.848 +/- 4.6653 | 0.42578125 |
| **T3 Beta** | 1.2064 +/- 2.2769 | 3.1013 +/- 4.6147 | 0.203125 |
| **T3 Delta** | 1.8074 +/- 4.4316 | 5.0052 +/- 8.848 | 0.25 |
| **T3 Gamma** | 0.74298 +/- 0.49325 | 1.5402 +/- 0.54273 | 0.00390625 |
| **T3 Overall** | 6.5382 +/- 6.6406 | 1.4862 +/- 6.833 | 0.07421875 |
| **O1 Theta** | -0.23403 +/- 10.9851 | -11.1431 +/- 29.825 | 0.5703125 |
| **O1 Alpha** | -0.23197 +/- 8.9813 | -8.1499 +/- 22.6267 | 0.8203125 |
| **O1 Beta** | -0.25094 +/- 7.3527 | -5.521 +/- 17.0123 | 0.65234375 |
| **O1 Delta** | -0.82876 +/- 15.3016 | -15.702 +/- 40.5947 | 0.65234375 |
| **O1 Gamma** | 0.40165 +/- 1.2843 | 0.8097 +/- 1.0279 | 0.30078125 |
| **O1 Overall** | 6.498 +/- 7.8361 | 1.0328 +/- 5.0457 | 0.02734375 |
| **R hemi Theta** | 2.3415 +/- 9.4861 | -8.8904 +/- 17.0788 | 0.01171875 |
| **R hemi Alpha** | 1.8234 +/- 7.7138 | -6.3935 +/- 12.4574 | 0.01171875 |
| **R hemi Beta** | 0.67096 +/- 6.8253 | -5.9457 +/- 12.1203 | 0.0390625 |
| **R hemi Delta** | 3.0104 +/- 10.6769 | -13.1478 +/- 25.1151 | 0.01171875 |
| **R hemi Gamma** | 0.518 +/- 0.72423 | 0.44795 +/- 2.0258 | 0.65234375 |
| **R hemi Overall** | 4.6961 +/- 5.9565 | -0.36634 +/- 5.2516 | 0.12890625 |
| **L hemi Theta** | 3.1292 +/- 5.6164 | -2.052 +/- 9.0131 | 0.203125 |
| **L hemi Alpha** | 2.5741 +/- 4.4679 | -1.3331 +/- 6.9008 | 0.1640625 |
| **L hemi Beta** | 1.8297 +/- 3.3136 | -0.45422 +/- 5.1272 | 0.42578125 |
| **L hemi Delta** | 3.6061 +/- 7.6206 | -3.1757 +/- 11.947 | 0.359375 |
| **L hemi Gamma** | 0.57999 +/- 0.66692 | 1.2553 +/- 0.52099 | 0.0390625 |
| **L hemi Overall** | 5.9472 +/- 7.119 | -0.37096 +/- 4.5773 | 0.0078125 |
| **Overall Theta** | 2.7354 +/- 6.3963 | -5.4713 +/- 11.7355 | 0.00390625 |
| **Overall Alpha** | 2.1987 +/- 5.0969 | -3.8634 +/- 8.6161 | 0.00390625 |
| **Overall Beta** | 1.2503 +/- 4.1183 | -3.2001 +/- 7.5022 | 0.01953125 |
| **Overall Delta** | 3.3082 +/- 7.8281 | -8.1617 +/- 16.6331 | 0.01953125 |
| **Overall Gamma** | 0.54899 +/- 0.65581 | 0.85162 +/- 0.98688 | 0.25 |
| **Overall Overall** | 5.3216 +/- 5.6374 | -0.36864 +/- 4.8508 | 0.0078125 |

**Table S13** Hemispheric asymmetry, overall and within each band, baseline & at 10-14 months follow-up

|  | **T1** | **T2** | **p** |
| --- | --- | --- | --- |
| **Frontal Theta** | 20.5772 +/- 38.9517 | 29.2669 +/- 85.3832 | 0.734375 |
| **Frontal Alpha** | 16.5126 +/- 32.4344 | 20.5524 +/- 59.4752 | 0.734375 |
| **Frontal Beta** | 13.6597 +/- 29.391 | 16.8664 +/- 49.164 | 0.8203125 |
| **Frontal Delta** | 24.1843 +/- 42.5084 | 39.0586 +/- 113.1497 | 0.734375 |
| **Frontal Gamma** | 0.84471 +/- 2.3337 | 0.70011 +/- 2.1053 | 0.91015625 |
| **Frontal Overall** | 2.4107 +/- 6.7521 | -1.6299 +/- 2.36 | 0.203125 |
| **Parietal Theta** | -12.0295 +/- 22.4849 | 9.0183 +/- 49.6928 | 0.203125 |
| **Parietal Alpha** | -9.1428 +/- 15.8184 | 7.3975 +/- 37.2695 | 0.203125 |
| **Parietal Beta** | -5.9206 +/- 9.3467 | 10.5194 +/- 40.8074 | 0.203125 |
| **Parietal Delta** | -14.6563 +/- 28.2184 | 16.7202 +/- 78.2266 | 0.25 |
| **Parietal Gamma** | -0.27111 +/- 1.8251 | 2.7008 +/- 7.6395 | 0.8203125 |
| **Parietal Overall** | 0.84305 +/- 11.4315 | -0.96454 +/- 2.9371 | 0.30078125 |
| **Temporal Theta** | -2.2095 +/- 2.8392 | -3.6394 +/- 10.097 | 1 |
| **Temporal Alpha** | -1.715 +/- 2.284 | -3.0151 +/- 8.346 | 1 |
| **Temporal Beta** | -1.1502 +/- 1.9204 | -2.9565 +/- 8.2389 | 1 |
| **Temporal Delta** | -2.9526 +/- 3.7714 | -3.9746 +/- 12.0702 | 0.91015625 |
| **Temporal Gamma** | -0.14798 +/- 0.49796 | -0.62003 +/- 1.4566 | 0.65234375 |
| **Temporal Overall** | -0.54696 +/- 10.3585 | 0.40332 +/- 4.3865 | 0.91015625 |
| **Occipital Theta** | -3.187 +/- 7.2825 | -7.2918 +/- 45.7226 | 0.734375 |
| **Occipital Alpha** | -2.6519 +/- 5.8506 | -4.6933 +/- 35.3003 | 0.734375 |
| **Occipital Beta** | -1.954 +/- 4.5866 | -2.4631 +/- 28.864 | 0.8203125 |
| **Occipital Delta** | -4.1917 +/- 10.5661 | -11.9163 +/- 58.6561 | 0.91015625 |
| **Occipital Gamma** | -0.17763 +/- 0.49706 | 0.44862 +/- 2.7943 | 0.734375 |
| **Occipital Overall** | 2.2976 +/- 7.0828 | 2.1727 +/- 6.5121 | 1 |
| **Overall Theta** | 3.1507 +/- 35.6462 | 27.3539 +/- 55.8528 | 0.1640625 |
| **Overall Alpha** | 3.0029 +/- 29.6704 | 20.2414 +/- 41.6988 | 0.30078125 |
| **Overall Beta** | 4.6349 +/- 27.5069 | 21.9667 +/- 44.0489 | 0.30078125 |
| **Overall Delta** | 2.3833 +/- 39.8042 | 39.888 +/- 83.9367 | 0.07421875 |
| **Overall Gamma** | 0.24797 +/- 1.8687 | 3.2295 +/- 8.8135 | 1 |
| **Overall Overall** | 5.0044 +/- 26.8923 | -0.018507 +/- 6.8576 | 0.734375 |

**Table S14** Inter-electrode coherence measures in the overall spectrum, baseline & at 10-14 months follow-up

|  | **T1** | **T2** | **p** |
| --- | --- | --- | --- |
| **Fp2-C4 Overall** | 0.35477 +/- 0.15703 | 0.40083 +/- 0.12923 | 0.5703125 |
| **Fp2-O2 Overall** | 0.32164 +/- 0.1431 | 0.36869 +/- 0.12188 | 0.49609375 |
| **Fp2-T4 Overall** | 0.31634 +/- 0.16253 | 0.29374 +/- 0.11374 | 0.8203125 |
| **Fp2-Fp1 Overall** | 0.34973 +/- 0.19698 | 0.3294 +/- 0.12962 | 0.734375 |
| **Fp2-C3 Overall** | 0.27815 +/- 0.14471 | 0.37251 +/- 0.21024 | 0.359375 |
| **Fp2-T3 Overall** | 0.26181 +/- 0.11675 | 0.29885 +/- 0.12541 | 0.42578125 |
| **Fp2-O1 Overall** | 0.28044 +/- 0.11633 | 0.35521 +/- 0.16382 | 0.203125 |
| **C4-O2 Overall** | 0.33756 +/- 0.18097 | 0.38909 +/- 0.14469 | 0.49609375 |
| **C4-T4 Overall** | 0.41244 +/- 0.22931 | 0.43054 +/- 0.1284 | 0.8203125 |
| **C4-Fp1 Overall** | 0.304 +/- 0.18818 | 0.29446 +/- 0.12835 | 0.65234375 |
| **C4-C3 Overall** | 0.32082 +/- 0.21856 | 0.32113 +/- 0.13536 | 0.5703125 |
| **C4-T3 Overall** | 0.30128 +/- 0.20448 | 0.26636 +/- 0.11066 | 0.91015625 |
| **C4-O1 Overall** | 0.29764 +/- 0.21828 | 0.33166 +/- 0.15027 | 0.359375 |
| **O2-T4 Overall** | 0.46206 +/- 0.25766 | 0.43837 +/- 0.17148 | 0.8203125 |
| **O2-Fp1 Overall** | 0.31979 +/- 0.20687 | 0.34837 +/- 0.14969 | 0.42578125 |
| **O2-C3 Overall** | 0.3281 +/- 0.2238 | 0.35242 +/- 0.18069 | 0.49609375 |
| **O2-T3 Overall** | 0.29867 +/- 0.18265 | 0.31411 +/- 0.14591 | 0.65234375 |
| **O2-O1 Overall** | 0.36089 +/- 0.22408 | 0.48892 +/- 0.20151 | 0.09765625 |
| **T4-Fp1 Overall** | 0.30555 +/- 0.21769 | 0.30833 +/- 0.16919 | 0.65234375 |
| **T4-C3 Overall** | 0.29162 +/- 0.24822 | 0.29937 +/- 0.1684 | 0.49609375 |
| **T4-T3 Overall** | 0.31163 +/- 0.21938 | 0.29677 +/- 0.12169 | 0.5703125 |
| **T4-O1 Overall** | 0.28724 +/- 0.19452 | 0.36347 +/- 0.1815 | 0.49609375 |
| **Fp1-C3 Overall** | 0.42314 +/- 0.18381 | 0.35939 +/- 0.10884 | 0.359375 |
| **Fp1-T3 Overall** | 0.31857 +/- 0.17718 | 0.2761 +/- 0.08605 | 0.8203125 |
| **Fp1-O1 Overall** | 0.40812 +/- 0.19359 | 0.39073 +/- 0.17367 | 0.91015625 |
| **C3-T3 Overall** | 0.39156 +/- 0.18079 | 0.46855 +/- 0.1637 | 0.42578125 |
| **C3-O1 Overall** | 0.41816 +/- 0.23737 | 0.4012 +/- 0.20474 | 1 |
| **T3-O1 Overall** | 0.40221 +/- 0.16023 | 0.43268 +/- 0.14813 | 0.734375 |


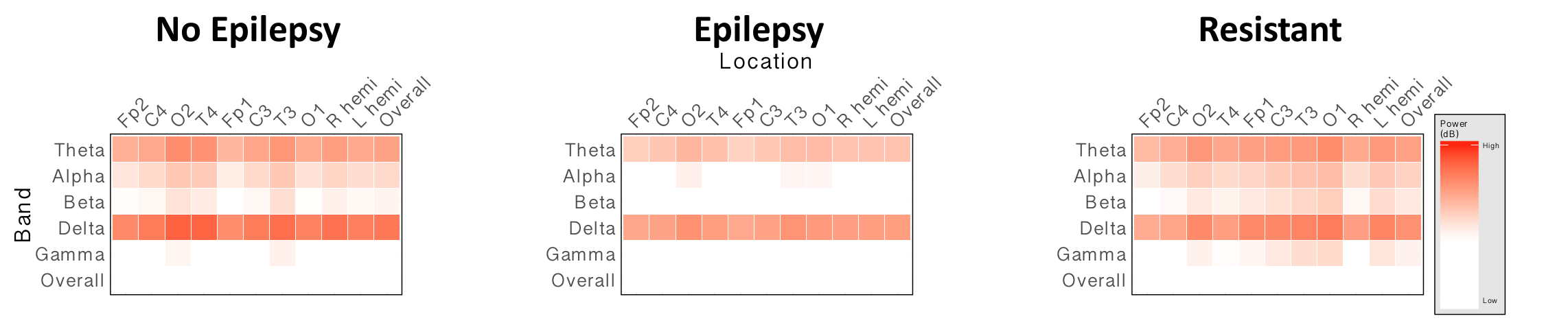


**Figure S9** Matrix of relative spectral power within each frequency band at each electrode location for No Epilepsy (*left*), Epilepsy (*middle*) and Resistant (*right*) groups. Each column represents a specific electrode location. Each row represents a frequency band. The intensity of each cell represents the relative power at the corresponding location and frequencies, with white representing low power and red representing high power. All groups were plotted using the same colour mapping, calculated based on maximum and minimum spectral power across the whole population, allowing direct comparison of intensities. Although there appears to be a trend towards differences in overall power distribution, these differences were not statistically significant (p < 0.05, Kruskal-Wallis H test)

**
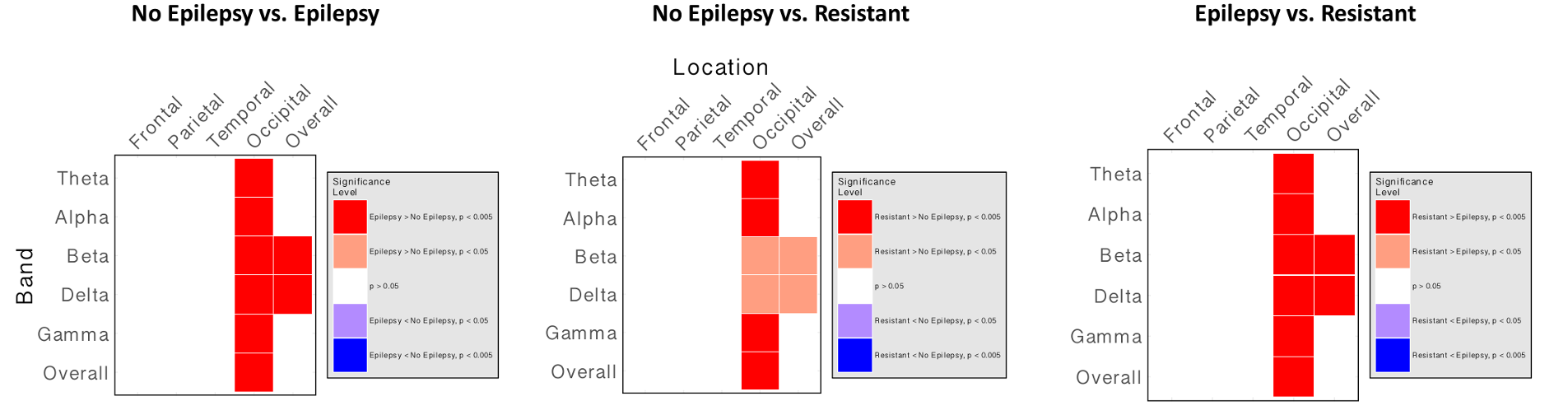
**

**Figure S10** Matrices illustrating the direction and magnitude of differences in hemispheric asymmetry identified on Kruskal-Wallis testing in pairwise comparisons between No Epilepsy, Epilepsy and Resistant groups. Each column represents a scalp location. Each row represents a frequency band. Whited-out cells were not identified as differing between epilepsy groups on Kruskal-Wallis testing and were therefore not included in pairwise comparisons. Of those compared, cell colour indicates the group with greater magnitude. Cell intensity indicates the statistical threshold crossed in pairwise comparison (Mann-Whitney U test). Comparisons between No Epilepsy and Epilepsy (*left*); No Epilepsy and Resistant (*middle*); and Epilepsy and Resistant (*right*) demonstrate a pattern of increasing left hemispheric predominance, particularly in occipital regions, with increasing severity of epilepsy status, suggesting potential utility of power distribution in occipital regions as a marker of epilepsy severity and treatment resistance.

**Table S15**  Overall power spectrum at each electrode location, No Epilepsy vs. Epilepsy vs. Resistant Epilepsy

|  | **Control** | **Epilepsy** | **Resistant** | **p** |
| --- | --- | --- | --- | --- |
| **Fp2** | 2.6128 +/- 9.7 | -1.0922 +/- 10.1889 | 2.8683 +/- 7.4955 | 0.285996277 |
| **C4** | 3.6559 +/- 10.0155 | -1.106 +/- 11.9987 | 3.5688 +/- 8.9701 | 0.233273962 |
| **T4** | 6.1802 +/- 9.8018 | 1.6782 +/- 10.5438 | 5.9592 +/- 8.758 | 0.163147619 |
| **O2** | 5.4172 +/- 9.822 | -0.61322 +/- 9.8444 | 4.8009 +/- 9.0333 | 0.109128233 |
| **Fp1** | 1.6154 +/- 9.4524 | -1.4384 +/- 9.4502 | 5.9721 +/- 11.7822 | 0.218318052 |
| **C3** | 4.0309 +/- 10.3003 | -1.0167 +/- 9.9833 | 6.4594 +/- 10.5804 | 0.151292108 |
| **T3** | 6.5271 +/- 9.9745 | 1.4763 +/- 9.1811 | 7.6653 +/- 9.9504 | 0.210444831 |
| **O1** | 2.9172 +/- 9.4864 | 0.16698 +/- 8.9874 | 8.2154 +/- 11.3589 | 0.197854325 |
| **R hemi** | 4.4665 +/- 9.2153 | -0.2833 +/- 9.8073 | 4.2993 +/- 8.4869 | 0.237225089 |
| **L hemi** | 3.7727 +/- 9.112 | -0.20296 +/- 9.0526 | 7.0781 +/- 9.5871 | 0.110366372 |
| **Overall** | 4.1196 +/- 8.9346 | -0.24316 +/- 9.2195 | 5.6887 +/- 8.7755 | 0.156345126 |

**Table S16** Power within each band at each electrode location, No Epilepsy vs. Epilepsy vs. Resistant Epilepsy

|  | **Control** | **Epilepsy** | **Resistant** | **p** |
| --- | --- | --- | --- | --- |
| **Fp2 Theta** | 1.7124 +/- 5.8386 | 5.8461 +/- 27.2672 | -11.3128 +/- 39.8916 | 0.349269901 |
| **Fp2 Alpha** | 1.6903 +/- 4.6335 | 5.0643 +/- 22.04 | -9.6753 +/- 33.8328 | 0.375996816 |
| **Fp2 Beta** | 1.3392 +/- 3.1438 | 3.8557 +/- 15.698 | -8.9824 +/- 31.2805 | 0.428959977 |
| **Fp2 Delta** | 1.6218 +/- 7.1847 | 6.9008 +/- 33.6494 | -11.9784 +/- 42.4782 | 0.37474973 |
| **Fp2 Gamma** | 2.6128 +/- 9.7 | -1.0922 +/- 10.1889 | 2.8683 +/- 7.4955 | 0.285996277 |
| **C4 Theta** | 2.242 +/- 9.446 | -2.3934 +/- 22.9373 | 5.2602 +/- 6.7297 | 0.14013959 |
| **C4 Alpha** | 2.146 +/- 7.7822 | -1.5243 +/- 16.588 | 4.7262 +/- 5.9815 | 0.150172333 |
| **C4 Beta** | 1.2159 +/- 4.9478 | -0.91241 +/- 10.8345 | 3.9134 +/- 4.5331 | 0.117151471 |
| **C4 Delta** | 1.8628 +/- 11.6499 | -3.1246 +/- 27.3181 | 5.7818 +/- 7.5272 | 0.124983604 |
| **C4 Gamma** | 0.76921 +/- 0.78846 | 1.7945 +/- 2.3966 | 0.96516 +/- 0.47764 | 0.244830579 |
| **T4 Theta** | 2.5866 +/- 27.7668 | -9.504 +/- 36.9143 | 0.90007 +/- 7.0388 | 0.227612061 |
| **T4 Alpha** | 2.7958 +/- 20.7834 | -6.7222 +/- 27.2615 | 1.0057 +/- 5.1209 | 0.249191181 |
| **T4 Beta** | 1.9382 +/- 14.9238 | -4.9896 +/- 20.0318 | 0.72092 +/- 4.605 | 0.192269804 |
| **T4 Delta** | 3.4865 +/- 38.4973 | -11.6797 +/- 42.9084 | 1.0271 +/- 7.753 | 0.278186931 |
| **T4 Gamma** | 0.75695 +/- 1.2324 | 1.7128 +/- 3.2336 | 0.69025 +/- 0.81215 | 0.585372639 |
| **O2 Theta** | -0.038889 +/- 5.1793 | -2.5277 +/- 10.9734 | 3.1652 +/- 3.6526 | 0.09095183 |
| **O2 Alpha** | 0.37718 +/- 3.9314 | -1.6303 +/- 7.8293 | 2.6935 +/- 2.7927 | 0.09734182 |
| **O2 Beta** | 0.56598 +/- 2.5567 | -0.98324 +/- 5.0833 | 2.3181 +/- 2.0117 | 0.059050334 |
| **O2 Delta** | -0.58262 +/- 6.9918 | -3.4334 +/- 13.0517 | 3.4651 +/- 4.3597 | 0.107986169 |
| **O2 Gamma** | 1.2683 +/- 0.63813 | 1.146 +/- 1.7667 | 0.93281 +/- 0.38973 | 0.481888088 |
| **Fp1 Theta** | 0.61021 +/- 11.1159 | 0.40806 +/- 16.7477 | -0.85153 +/- 8.066 | 0.7291554 |
| **Fp1 Alpha** | 0.9861 +/- 8.5264 | 0.4024 +/- 12.0349 | -0.31585 +/- 5.9418 | 0.737564311 |
| **Fp1 Beta** | 0.53487 +/- 5.7528 | 0.47647 +/- 7.8223 | -0.073357 +/- 4.9246 | 0.796297522 |
| **Fp1 Delta** | -0.28223 +/- 15.1344 | 0.27977 +/- 21.1297 | -1.7149 +/- 11.0569 | 0.732696701 |
| **Fp1 Gamma** | 0.95737 +/- 1.7163 | 1.8167 +/- 1.5705 | 1.0993 +/- 0.89081 | 0.343214967 |
| **C3 Theta** | -53.2298 +/- 139.0573 | -5.9261 +/- 18.8045 | 2.8867 +/- 4.5105 | 0.450067671 |
| **C3 Alpha** | -41.1018 +/- 110.5564 | -4.0043 +/- 13.4685 | 2.6551 +/- 3.8404 | 0.460837726 |
| **C3 Beta** | -24.0198 +/- 69.0413 | -2.2045 +/- 8.8398 | 2.411 +/- 3.0441 | 0.425785067 |
| **C3 Delta** | -69.487 +/- 181.5166 | -7.3991 +/- 22.6354 | 3.2467 +/- 5.268 | 0.52703896 |
| **C3 Gamma** | 4.4095 +/- 12.3343 | 1.406 +/- 2.3496 | 1.0924 +/- 0.37859 | 0.654285837 |
| **T3 Theta** | 6.4707 +/- 12.7704 | 1.4944 +/- 4.6833 | 1.0126 +/- 5.1015 | 0.77366103 |
| **T3 Alpha** | 5.3312 +/- 10.3556 | 1.3436 +/- 3.7543 | 0.89051 +/- 4.417 | 0.692589868 |
| **T3 Beta** | 4.2325 +/- 7.5442 | 1.1248 +/- 2.8879 | 0.88577 +/- 3.4568 | 0.770934898 |
| **T3 Delta** | 8.619 +/- 17.6476 | 1.5523 +/- 6.0518 | 1.0983 +/- 5.7795 | 0.745880881 |
| **T3 Gamma** | 0.91321 +/- 0.626 | 0.93621 +/- 0.60183 | 0.84985 +/- 0.59623 | 0.859172369 |
| **O1 Theta** | -4.9256 +/- 14.2189 | 0.0077613 +/- 10.1475 | 1.7278 +/- 2.4334 | 0.588392076 |
| **O1 Alpha** | -3.2189 +/- 10.4893 | -0.11845 +/- 8.2784 | 1.652 +/- 1.9474 | 0.63397272 |
| **O1 Beta** | -1.4857 +/- 6.7856 | -0.056828 +/- 6.5877 | 1.6094 +/- 1.4979 | 0.649473164 |
| **O1 Delta** | -6.8683 +/- 19.1154 | -0.60702 +/- 13.1824 | 1.7915 +/- 2.9818 | 0.593792383 |
| **O1 Gamma** | 1.6287 +/- 1.5576 | 0.69772 +/- 1.3236 | 1.0157 +/- 0.31416 | 0.373817131 |
| **R hemi Theta** | 1.6256 +/- 6.6872 | -2.1448 +/- 11.6925 | -0.49674 +/- 8.7227 | 0.338496662 |
| **R hemi Alpha** | 1.7523 +/- 4.932 | -1.2031 +/- 8.16 | -0.31243 +/- 7.1717 | 0.325019692 |
| **R hemi Beta** | 1.2648 +/- 3.8059 | -0.75742 +/- 5.6239 | -0.50746 +/- 6.846 | 0.279267267 |
| **R hemi Delta** | 1.5971 +/- 9.2529 | -2.8342 +/- 13.6166 | -0.42592 +/- 9.3754 | 0.289121018 |
| **R hemi Gamma** | 0.89844 +/- 0.47481 | 1.2814 +/- 1.1581 | 0.7177 +/- 0.62831 | 0.263341838 |
| **L hemi Theta** | -12.7686 +/- 38.019 | -1.004 +/- 6.1934 | 1.1939 +/- 3.9161 | 0.686576101 |
| **L hemi Alpha** | -9.5009 +/- 29.9311 | -0.59423 +/- 4.6353 | 1.2205 +/- 3.1585 | 0.62910623 |
| **L hemi Beta** | -5.1846 +/- 18.7712 | -0.16502 +/- 3.3103 | 1.2082 +/- 2.5014 | 0.55878237 |
| **L hemi Delta** | -17.0044 +/- 49.8648 | -1.5435 +/- 7.7075 | 1.1054 +/- 4.81 | 0.748380277 |
| **L hemi Gamma** | 1.9772 +/- 3.5764 | 1.2141 +/- 0.76071 | 1.0143 +/- 0.45003 | 0.810046578 |
| **Overall Theta** | -5.5715 +/- 19.5715 | -1.5744 +/- 8.5329 | 0.34859 +/- 5.0428 | 0.678466689 |
| **Overall Alpha** | -3.8742 +/- 15.134 | -0.89861 +/- 6.0351 | 0.45398 +/- 4.0726 | 0.678466689 |
| **Overall Beta** | -1.9599 +/- 9.5789 | -0.46122 +/- 4.1853 | 0.35036 +/- 3.7218 | 0.711605471 |
| **Overall Delta** | -7.7038 +/- 26.0814 | -2.1889 +/- 10.101 | 0.3397 +/- 5.6168 | 0.677621949 |
| **Overall Gamma** | 1.4378 +/- 1.8875 | 1.2478 +/- 0.92243 | 0.86599 +/- 0.51772 | 0.590499958 |

**Table S17** Hemispheric asymmetry, overall and within each band, No Epilepsy vs. Epilepsy vs. Resistant Epilepsy

|  | **Control** | **Epilepsy** | **Resistant** | **p** |
| --- | --- | --- | --- | --- |
| **Frontal Theta** | -1.1022 +/- 7.8211 | -5.438 +/- 36.3356 | 10.4616 +/- 42.3659 | 0.983684762 |
| **Frontal Alpha** | -0.70413 +/- 5.7692 | -4.662 +/- 28.3331 | 9.3596 +/- 35.686 | 0.987823905 |
| **Frontal Beta** | -0.80429 +/- 3.975 | -3.3793 +/- 19.6041 | 8.9091 +/- 32.7738 | 0.928745744 |
| **Frontal Delta** | -1.9041 +/- 11.0206 | -6.6207 +/- 44.7755 | 10.2637 +/- 46.0064 | 0.993125437 |
| **Frontal Gamma** | 0.15808 +/- 1.2214 | 1.3442 +/- 4.1404 | 0.81674 +/- 1.4801 | 0.949961788 |
| **Frontal Overall** | -0.99737 +/- 3.5989 | -0.34629 +/- 6.3605 | 3.1038 +/- 9.2391 | 0.306343809 |
| **Parietal Theta** | -55.4714 +/- 144.3855 | -3.5328 +/- 19.3571 | -2.3736 +/- 5.3177 | 0.63485092 |
| **Parietal Alpha** | -43.2475 +/- 115.2657 | -2.48 +/- 13.8484 | -2.0711 +/- 4.8929 | 0.765405458 |
| **Parietal Beta** | -25.2363 +/- 71.6456 | -1.292 +/- 8.3432 | -1.5024 +/- 3.5523 | 0.556107299 |
| **Parietal Delta** | -71.35 +/- 187.3084 | -4.2744 +/- 24.2359 | -2.535 +/- 5.7098 | 0.649832883 |
| **Parietal Gamma** | 3.6402 +/- 12.6777 | -0.38849 +/- 1.4324 | 0.12723 +/- 0.28942 | 0.296244614 |
| **Parietal Overall** | 0.37501 +/- 4.8318 | 0.089401 +/- 7.818 | 2.8907 +/- 6.7713 | 0.443286848 |
| **Temporal Theta** | 3.8841 +/- 30.4808 | 10.9982 +/- 37.6114 | 0.11257 +/- 2.1755 | 0.369103664 |
| **Temporal Alpha** | 2.5354 +/- 22.5851 | 8.0656 +/- 27.8551 | -0.11513 +/- 1.0199 | 0.378398676 |
| **Temporal Beta** | 2.2943 +/- 16.424 | 6.1144 +/- 20.5508 | 0.16479 +/- 1.3556 | 0.46622758 |
| **Temporal Delta** | 5.133 +/- 42.761 | 13.2323 +/- 43.8174 | 0.071114 +/- 2.3183 | 0.336371358 |
| **Temporal Gamma** | 0.15627 +/- 1.5927 | -0.77658 +/- 3.3365 | 0.15959 +/- 0.284 | 0.113897364 |
| **Temporal Overall** | 0.34676 +/- 6.053 | -0.20192 +/- 8.1077 | 1.7061 +/- 3.4623 | 0.767562873 |
| **Occipital Theta** | -4.8867 +/- 13.0961 | 2.5355 +/- 15.0287 | -1.4374 +/- 2.1564 | 0.182328988 |
| **Occipital Alpha** | -3.5961 +/- 10.0162 | 1.5118 +/- 11.1222 | -1.0415 +/- 1.5432 | 0.188862531 |
| **Occipital Beta** | -2.0516 +/- 6.2974 | 0.92641 +/- 7.7125 | -0.70875 +/- 0.97302 | 0.189534747 |
| **Occipital Delta** | -6.2856 +/- 17.3896 | 2.8263 +/- 18.1464 | -1.6736 +/- 2.5758 | 0.132172416 |
| **Occipital Gamma** | 0.36043 +/- 1.4827 | -0.44829 +/- 2.067 | 0.08291 +/- 0.21168 | 0.649832883 |
| **Occipital Overall** | -2.4999 +/- 5.8124 | 0.78025 +/- 4.5973 | 3.4145 +/- 6.0103 | 0.046499489 |
| **Overall Theta** | -57.5761 +/- 152.2186 | 4.5629 +/- 30.7018 | 6.7626 +/- 36.0273 | 0.601513892 |
| **Overall Alpha** | -45.0125 +/- 121.6025 | 2.4355 +/- 22.072 | 6.1318 +/- 30.0605 | 0.626773768 |
| **Overall Beta** | -25.7972 +/- 76.5926 | 2.3697 +/- 15.5461 | 6.8627 +/- 28.522 | 0.598579009 |
| **Overall Delta** | -74.4068 +/- 196.9102 | 5.1631 +/- 36.1128 | 6.1257 +/- 39.1669 | 0.484194968 |
| **Overall Gamma** | 4.315 +/- 13.7284 | -0.26918 +/- 2.6423 | 1.1865 +/- 1.3995 | 0.199799274 |
| **Overall Overall** | -2.7755 +/- 16.291 | 0.3215 +/- 16.1323 | 11.1151 +/- 17.8193 | 0.086754312 |

**Table S18** Percentage of total variance explained by each of the first five principal components of the coherence measures for each epilepsy group. P value represents value of statistical comparison of corresponding principal components using Kruskal-Wallis H test.

| **Principal Component** | **No Epilepsy** | **Epilepsy** | **Resistant** | **p** |
| --- | --- | --- | --- | --- |
| 1 | 51.42 | 50.45 | 42.41 | 0.04 |
| 2 | 11.15 | 8.86 | 17.89 | 0.86 |
| 3 | 8.83 | 8.09 | 14.06 | 0.01 |
| 4 | 6.17 | 6.52 | 11.86 | 0.35 |
| 5 | 4.9 | 5.88 | 5.76 | 0.48 |

**Table S19** Inter-electrode coherence measures in the overall spectrum, No Epilepsy vs. Epilepsy vs. Resistant Epilepsy

|  | **Control** | **Epilepsy** | **Resistant** | **p** |
| --- | --- | --- | --- | --- |
| **Fp2-C4** | 0.35781 +/- 0.15408 | 0.37924 +/- 0.19272 | 0.43995 +/- 0.16871 | 0.562494595 |
| **Fp2-O2** | 0.37851 +/- 0.18893 | 0.36023 +/- 0.18167 | 0.46745 +/- 0.21122 | 0.428692851 |
| **Fp2-T4** | 0.28902 +/- 0.11479 | 0.29342 +/- 0.14875 | 0.40968 +/- 0.16268 | 0.211759833 |
| **Fp2-Fp1** | 0.37966 +/- 0.13396 | 0.42464 +/- 0.18162 | 0.54612 +/- 0.21806 | 0.151173478 |
| **Fp2-C3** | 0.32052 +/- 0.15072 | 0.37284 +/- 0.17867 | 0.40302 +/- 0.14353 | 0.370622572 |
| **Fp2-T3** | 0.31704 +/- 0.14214 | 0.29244 +/- 0.13808 | 0.38656 +/- 0.1731 | 0.344547839 |
| **Fp2-O1** | 0.32382 +/- 0.13295 | 0.33579 +/- 0.13907 | 0.41932 +/- 0.13937 | 0.212891411 |
| **C4-O2** | 0.35011 +/- 0.16172 | 0.37829 +/- 0.15532 | 0.41997 +/- 0.1845 | 0.663145784 |
| **C4-T4** | 0.38936 +/- 0.23516 | 0.3649 +/- 0.20377 | 0.39877 +/- 0.1513 | 0.618613815 |
| **C4-Fp1** | 0.29694 +/- 0.15781 | 0.37192 +/- 0.16854 | 0.3976 +/- 0.13738 | 0.113881599 |
| **C4-C3** | 0.37865 +/- 0.19115 | 0.42996 +/- 0.18775 | 0.4588 +/- 0.20106 | 0.512683243 |
| **C4-T3** | 0.31849 +/- 0.16451 | 0.29937 +/- 0.1314 | 0.33607 +/- 0.10636 | 0.568889625 |
| **C4-O1** | 0.32435 +/- 0.18692 | 0.32685 +/- 0.13027 | 0.39309 +/- 0.17854 | 0.432507668 |
| **O2-T4** | 0.47156 +/- 0.24865 | 0.3862 +/- 0.22072 | 0.46425 +/- 0.14497 | 0.304160628 |
| **O2-Fp1** | 0.33877 +/- 0.16656 | 0.37121 +/- 0.12907 | 0.4341 +/- 0.14216 | 0.191826725 |
| **O2-C3** | 0.33195 +/- 0.19199 | 0.386 +/- 0.16103 | 0.38804 +/- 0.13662 | 0.512434911 |
| **O2-T3** | 0.34121 +/- 0.16553 | 0.30513 +/- 0.13441 | 0.35005 +/- 0.12555 | 0.760231322 |
| **O2-O1** | 0.43936 +/- 0.19216 | 0.45424 +/- 0.18308 | 0.48025 +/- 0.212 | 0.769957265 |
| **T4-Fp1** | 0.28447 +/- 0.14521 | 0.32872 +/- 0.16316 | 0.40242 +/- 0.1605 | 0.189351178 |
| **T4-C3** | 0.28185 +/- 0.18783 | 0.32836 +/- 0.17341 | 0.31576 +/- 0.10422 | 0.342376643 |
| **T4-T3** | 0.32568 +/- 0.17451 | 0.30068 +/- 0.14388 | 0.40317 +/- 0.10988 | 0.125165398 |
| **T4-O1** | 0.34336 +/- 0.16842 | 0.30096 +/- 0.12721 | 0.43758 +/- 0.17425 | 0.191340517 |
| **Fp1-C3** | 0.3764 +/- 0.17022 | 0.44152 +/- 0.19978 | 0.48089 +/- 0.079988 | 0.170273581 |
| **Fp1-T3** | 0.33372 +/- 0.1626 | 0.30536 +/- 0.13131 | 0.395 +/- 0.13578 | 0.239159456 |
| **Fp1-O1** | 0.41025 +/- 0.18807 | 0.37577 +/- 0.14418 | 0.48643 +/- 0.10757 | 0.141279049 |
| **C3-T3** | 0.49944 +/- 0.20533 | 0.34733 +/- 0.16608 | 0.40843 +/- 0.14616 | 0.05106204 |
| **C3-O1** | 0.41491 +/- 0.19589 | 0.42801 +/- 0.14423 | 0.46752 +/- 0.14457 | 0.637522242 |
| **T3-O1** | 0.49342 +/- 0.19156 | 0.37146 +/- 0.12831 | 0.45744 +/- 0.14224 | 0.152497617 |

**Table S20** Hemispheric asymmetry, all epilepsy vs. No epilepsy

|  | **No Epilepsy** | **All Epilepsy** | **p** |
| --- | --- | --- | --- |
| **Frontal Theta** | -1.1022 +/- 7.8211 | -0.13815 +/- 38.2875 | 0.908941381 |
| **Frontal Alpha** | -0.70413 +/- 5.7692 | 0.011883 +/- 30.9308 | 0.888825633 |
| **Frontal Beta** | -0.80429 +/- 3.975 | 0.71688 +/- 24.7501 | 0.888825633 |
| **Frontal Delta** | -1.9041 +/- 11.0206 | -0.9926 +/- 44.9198 | 0.929115687 |
| **Frontal Gamma** | 0.15808 +/- 1.2214 | 1.1684 +/- 3.4513 | 0.908941381 |
| **Frontal Overall** | -0.99737 +/- 3.5989 | 0.80374 +/- 7.4245 | 0.280053927 |
| **Parietal Theta** | -55.4714 +/- 144.3855 | -3.1464 +/- 15.9149 | 0.750708449 |
| **Parietal Alpha** | -43.2475 +/- 115.2657 | -2.3437 +/- 11.5064 | 0.969588304 |
| **Parietal Beta** | -25.2363 +/- 71.6456 | -1.3622 +/- 7.0177 | 0.828956848 |
| **Parietal Delta** | -71.35 +/- 187.3084 | -3.6946 +/- 19.8418 | 0.848820802 |
| **Parietal Gamma** | 3.6402 +/- 12.6777 | -0.21658 +/- 1.1938 | 0.173899895 |
| **Parietal Overall** | 0.37501 +/- 4.8318 | 1.0232 +/- 7.4589 | 0.258042017 |
| **Temporal Theta** | 3.8841 +/- 30.4808 | 7.3697 +/- 30.8463 | 1 |
| **Temporal Alpha** | 2.5354 +/- 22.5851 | 5.3387 +/- 22.8443 | 0.908941381 |
| **Temporal Beta** | 2.2943 +/- 16.424 | 4.1312 +/- 16.8583 | 1 |
| **Temporal Delta** | 5.133 +/- 42.761 | 8.8453 +/- 35.9715 | 0.908941381 |
| **Temporal Gamma** | 0.15627 +/- 1.5927 | -0.46452 +/- 2.7364 | 0.190552622 |
| **Temporal Overall** | 0.34676 +/- 6.053 | 0.43409 +/- 6.8821 | 0.602341148 |
| **Occipital Theta** | -4.8867 +/- 13.0961 | 1.2112 +/- 12.3441 | 0.789567332 |
| **Occipital Alpha** | -3.5961 +/- 10.0162 | 0.6607 +/- 9.1056 | 0.770065725 |
| **Occipital Beta** | -2.0516 +/- 6.2974 | 0.38136 +/- 6.3009 | 0.989860585 |
| **Occipital Delta** | -6.2856 +/- 17.3896 | 1.3264 +/- 14.8819 | 0.674944965 |
| **Occipital Gamma** | 0.36043 +/- 1.4827 | -0.27122 +/- 1.6927 | 0.848820802 |
| **Occipital Overall** | -2.4999 +/- 5.8124 | 1.6583 +/- 5.1369 | 0.018722046 |
| **Overall Theta** | -57.5761 +/- 152.2186 | 5.2962 +/- 31.7946 | 0.366906443 |
| **Overall Alpha** | -45.0125 +/- 121.6025 | 3.6676 +/- 24.4113 | 0.353563397 |
| **Overall Beta** | -25.7972 +/- 76.5926 | 3.8673 +/- 20.2457 | 0.340531268 |
| **Overall Delta** | -74.4068 +/- 196.9102 | 5.4839 +/- 36.299 | 0.268893353 |
| **Overall Gamma** | 4.315 +/- 13.7284 | 0.21604 +/- 2.375 | 0.809201661 |
| **Overall Overall** | -2.7755 +/- 16.291 | 3.9194 +/- 17.1284 | 0.124123472 |

**Table S21** Hemispheric asymmetry, treatment resistant vs. Responsive epilepsy

|  | **Epilepsy** | **Resistant** | **p** |
| --- | --- | --- | --- |
| **Frontal Theta** | -5.438 +/- 36.3356 | 10.4616 +/- 42.3659 | 1 |
| **Frontal Alpha** | -4.662 +/- 28.3331 | 9.3596 +/- 35.686 | 0.975573691 |
| **Frontal Beta** | -3.3793 +/- 19.6041 | 8.9091 +/- 32.7738 | 0.87832485 |
| **Frontal Delta** | -6.6207 +/- 44.7755 | 10.2637 +/- 46.0064 | 0.87832485 |
| **Frontal Gamma** | 1.3442 +/- 4.1404 | 0.81674 +/- 1.4801 | 0.83028945 |
| **Frontal Overall** | -0.34629 +/- 6.3605 | 3.1038 +/- 9.2391 | 0.232428587 |
| **Parietal Theta** | -3.5328 +/- 19.3571 | -2.3736 +/- 5.3177 | 0.408405541 |
| **Parietal Alpha** | -2.48 +/- 13.8484 | -2.0711 +/- 4.8929 | 0.602703582 |
| **Parietal Beta** | -1.292 +/- 8.3432 | -1.5024 +/- 3.5523 | 0.283876475 |
| **Parietal Delta** | -4.2744 +/- 24.2359 | -2.535 +/- 5.7098 | 0.443994376 |
| **Parietal Gamma** | -0.38849 +/- 1.4324 | 0.12723 +/- 0.28942 | 0.443994376 |
| **Parietal Overall** | 0.089401 +/- 7.818 | 2.8907 +/- 6.7713 | 0.560732518 |
| **Temporal Theta** | 10.9982 +/- 37.6114 | 0.11257 +/- 2.1755 | 0.133533481 |
| **Temporal Alpha** | 8.0656 +/- 27.8551 | -0.11513 +/- 1.0199 | 0.133533481 |
| **Temporal Beta** | 6.1144 +/- 20.5508 | 0.16479 +/- 1.3556 | 0.187972492 |
| **Temporal Delta** | 13.2323 +/- 43.8174 | 0.071114 +/- 2.3183 | 0.133533481 |
| **Temporal Gamma** | -0.77658 +/- 3.3365 | 0.15959 +/- 0.284 | 0.168253317 |
| **Temporal Overall** | -0.20192 +/- 8.1077 | 1.7061 +/- 3.4623 | 0.736264025 |
| **Occipital Theta** | 2.5355 +/- 15.0287 | -1.4374 +/- 2.1564 | 0.070840479 |
| **Occipital Alpha** | 1.5118 +/- 11.1222 | -1.0415 +/- 1.5432 | 0.070840479 |
| **Occipital Beta** | 0.92641 +/- 7.7125 | -0.70875 +/- 0.97302 | 0.070840479 |
| **Occipital Delta** | 2.8263 +/- 18.1464 | -1.6736 +/- 2.5758 | 0.053734193 |
| **Occipital Gamma** | -0.44829 +/- 2.067 | 0.08291 +/- 0.21168 | 0.408405541 |
| **Occipital Overall** | 0.78025 +/- 4.5973 | 3.4145 +/- 6.0103 | 0.443994376 |
| **Overall Theta** | 4.5629 +/- 30.7018 | 6.7626 +/- 36.0273 | 0.602703582 |
| **Overall Alpha** | 2.4355 +/- 22.072 | 6.1318 +/- 30.0605 | 0.83028945 |
| **Overall Beta** | 2.3697 +/- 15.5461 | 6.8627 +/- 28.522 | 0.736264025 |
| **Overall Delta** | 5.1631 +/- 36.1128 | 6.1257 +/- 39.1669 | 0.690599165 |
| **Overall Gamma** | -0.26918 +/- 2.6423 | 1.1865 +/- 1.3995 | 0.08093937 |
| **Overall Overall** | 0.3215 +/- 16.1323 | 11.1151 +/- 17.8193 | 0.133533481 |
